# Meta-analysis of archived DNA microarrays identifies genes regulated by hypoxia and involved in a metastatic phenotype in cancer cells

**DOI:** 10.1186/1471-2407-10-176

**Published:** 2010-04-30

**Authors:** Michael Pierre, Benoît DeHertogh, Anthoula Gaigneaux, Bertrand DeMeulder, Fabrice Berger, Eric Bareke, Carine Michiels, Eric Depiereux

**Affiliations:** 1 Molecular Biology Research Unit (URBM), University of Namur - FUNDP, Namur, Belgium; 2 Cell Biology Research Unit (URBC), University of Namur - FUNDP, Namur, Belgium

## Abstract

**Background:**

Metastasis is a major cancer-related cause of death. Recent studies have described metastasis pathways. However, the exact contribution of each pathway remains unclear. Another key feature of a tumor is the presence of hypoxic areas caused by a lack of oxygen at the center of the tumor. Hypoxia leads to the expression of pro-metastatic genes as well as the repression of anti-metastatic genes. As many Affymetrix datasets about metastasis and hypoxia are publicly available and not fully exploited, this study proposes to re-analyze these datasets to extract new information about the metastatic phenotype induced by hypoxia in different cancer cell lines.

**Methods:**

Affymetrix datasets about metastasis and/or hypoxia were downloaded from GEO and ArrayExpress. AffyProbeMiner and GCRMA packages were used for pre-processing and the Window Welch *t *test was used for processing. Three approaches of meta-analysis were eventually used for the selection of genes of interest.

**Results:**

Three complementary approaches were used, that eventually selected 183 genes of interest. Out of these 183 genes, 99, among which the well known *JUNB*, *FOS *and *TP63*, have already been described in the literature to be involved in cancer. Moreover, 39 genes of those, such as *SERPINE1 *and *MMP7*, are known to regulate metastasis. Twenty-one genes including *VEGFA *and *ID2 *have also been described to be involved in the response to hypoxia. Lastly, DAVID classified those 183 genes in 24 different pathways, among which 8 are directly related to cancer while 5 others are related to proliferation and cell motility. A negative control composed of 183 random genes failed to provide such results. Interestingly, 6 pathways retrieved by DAVID with the 183 genes of interest concern pathogen recognition and phagocytosis.

**Conclusion:**

The proposed methodology was able to find genes actually known to be involved in cancer, metastasis and hypoxia and, thus, we propose that the other genes selected based on the same methodology are of prime interest in the metastatic phenotype induced by hypoxia.

## Background

One of the major causes of death by cancer is metastasis. Determining the mechanisms of metastasis initiation and growth should thus improve therapy. Cancer cells have developed many mechanisms to detach from the primary tumor, invade surrounding tissues, migrate and colonize distant organs. These mechanisms include changes in cell-cell and cell-matrix adhesion molecules, extracellular matrix degradation enzymes, cytoskeleton regulation factors and cell-cell communication through cytokines, for example [1]. Recently, high-throughput studies performed in several cancer cell lines identified specific metastasis pathways [2]. However, the exact contribution of these pathways in cell migration and tissue invasion still remains unclear.

Another key feature of a tumor is the presence of hypoxic areas. Hypoxic areas within a tumor are the result of the progressively increasing distance between cells and blood vessels as the tumor is growing, as well as of the abnormal new vasculature. Tumor hypoxia is a marker for poor prognosis. Several hypotheses have been proposed to explain this observation: (i) hypoxia initiates adaptation mediated by the transcription factor HIF-1 which enhances cancer cell survival [3]; (ii) this adaptation also triggers the angiogenesis process; (iii) hypoxia leads to less effective radiotherapy and chemotherapy [4] and (iv) more and more experimental data suggest that hypoxia improves metastasis and/or selects cancer cells with high metastatic potential [5]. Changes in gene expression induced by hypoxia and leading to a migratory and invasive phenotype of tumor cells have been identified. The genes involved code for example for cadherins, plasminogen activators and their receptors, and matrix metalloproteinases [6].

DNA microarrays appeared more than a decade ago and are now one of the most common tools in many molecular biology and medical laboratories. The technique allows the assessment of transcript levels for thousands of genes in one single experiment. Nowadays, thanks to progress in sequencing, an entire genome can be represented on one single microarray [7,8]. In short, a microarray is a physical support to which oligonucleotides (the probes) representing the genes are attached. Then, the labelled mRNAs from a biological sample are incubated with the microarray, thus enabling the labelled mRNAs to hybridize to the probes. The signal is then scanned and several experimental conditions are compared to determine which genes are differentially expressed under a particular condition. Microarrays can differ in the way the probes are attached to the surface, in the length of the probes and in the number of probes per gene, but the principle is always the same.

Affymetrix GeneChips are the most popular type of microarrays. Affymetrix GeneChips display probes that are synthetized *in situ *by photolithography [9]. Typically, each gene in a genome is represented by 11 to 20 perfect match probes of 25 nucleotides and by the same number of mismatch probes differing only in the central nucleotide [10].

Since their release, Affymetrix GeneChips and DNA microarrays in general have encountered many difficulties. The first derives from the fact that if there is only one replicate per condition, no statistical test can be performed at the gene level. Results thus only rely on ratio (fold change) between expression values obtained from different conditions [11]. On the other hand, when several replicates are available, the question can be addressed from a statistical point of view [12]. As one statistical test is performed for each gene, thousands of tests are performed on a single array. This leads to a very large number of false positives (genes detected as differentially expressed when they are not) and false negatives (genes undetected when they are actually differentially expressed), compromising interpretation of the results [13]. When applied, a correction for multiple testing decreases the threshold of significance at such a low level that the number of false negatives increases dramatically. Many statistical methods have recently emerged to solve this problem, from the classical Student *t*-test [14] to more sophisticated tests which fine-tune the estimate of gene variance [15-20]. However, the problem still remains fundamentally unsolved and a large number of expensive replicates are needed to gain in positive and negative predictive power.

Another issue tackled over the past few years concerns the Chip Definition File (CDF), which is involved upstream of the statistical analysis. A CDF is a file developed by Affymetrix that links several probes (probe set) to a given gene name. The probes representing the gene reflect the status of genomic databases several years ago. Since then, genomic information and thus the arguments used to assign given probes to a given probe set have evolved and "alternative" CDFs have emerged [21-23].

Other steps before the statistical processing itself concern pre-processing of the data. Pre-processing steps include background correction within each array of a same experiment, normalization (rescaling) of values between replicates [24-26] and summarization of probe values to obtain a unique expression value per probe set [27]. Scores of methods are available for each pre-processing step [28,29] and a combination of these methods can potentially generate thousands of pre-processings.

A technique's lack of biological reproducibility is not the least of the problems encountered in DNA microarray experiments [30]. Few genes are common to the top gene lists from several experiments, even between sub-samples of a same large dataset. Due to the small number of replicates generally used in an experiment and the very large number of genes tested, varying variance estimates lead to differences in the p values associated with any statistical test such that a given gene can randomly move from the first to the thousandth place in the top list.

The fact that there are numerous methods which continue to evolve, combined with the observation of unstable results, constitutes an attractive challenge which can now be tackled thanks to the emergence of public databases such as Gene Expression Omnibus (GEO) [31] and ArrayExpress [32] which collect millions of pieces of expression data. Datasets can be reanalysed from scratch with new parameters (including alternative CDFs) and by combining several datasets relative to the same biological question in one same analysis.

In this study we have tried to find genes, and possibly pathways, that were not previously known to be involved in the metastasis induced by hypoxia. The objective of this paper is to increase our understanding of the disease using new methods to exploit the huge amount of DNA microarrays publicly available. Since the mechanisms underlying the metastatic phenotype are identical in every cancer cell type [33], this work considered all datasets on metastasis, regardless of the cancer cell type. Our methodology combines well-known published steps from classical analysis with new approaches to analyze several datasets at once. A similar study performed at a smaller scale on breast cancer has been published recently [34], thus validating the feasibility of this type of approach.

## Methods

### Datasets

All the datasets used in this study were downloaded from two databases (ArrayExpress [32] and Gene Expression Omnibus [31]) and are all generated with Affymetrix platforms. Most of the raw data in .CEL files format were publicly available. If not, the authors were contacted directly. Datasets containing more than one GeneChip model and/or containing more than two conditions were split into sub-datasets. Table 1 presents detailed information about these datasets.

**Table 1:** The datasets retrieved from GEO and ArrayExpress

Data set accession numbers	GeneChip models	Databases	Availability	Experimental conditions
E-GEOD-1323	HG-U133A	AE	Available	3 human colorectal cancer derived from a primary tumor VS. 3 corresponding lymph node metastases

E-GEOD-2280	HG-U133A	AE	Available	8 squamous cell carcinoma of the oral cavity VS. 19 corresponding lymph node metastases

E-MEXP-44	HG-U95Av2	AE	Available	15 head and neck squamous cell carcinoma VS. 3 corresponding lymph node metastases
	
	HG-UgeneFL	AE	Available	12 head and neck squamous cell carcinoma VS. 11 corresponding lymph node metastases

GSE1056	HG-U95Av2	GEO	Not available	2 human hepatocellular carcinoma under hypoxia for 2 hours VS. 2 control human hepatocellular carcinoma
	
	HG-U95Av2	GEO	Not available	2 human hepatocellular carcinoma under hypoxia for 24 hours VS. 2 control human hepatocellular carcinoma

GSE2280	HG-U133A	GEO	Available	22 squamous cell carcinoma of the oral cavity VS. 5 corresponding lymph node metastases

GSE2603	HG-U133A	GEO	Available	100 primary breast cancer VS. 21 lung metastases

GSE3325	HG-U133Plus2.0	GEO	Available	7 primary prostate cancer VS. 6 metastases

GSE4086	HG-U133Plus2.0	GEO	Available	2 human Burkitt's lymphoma under hypoxia VS. 2 control human Burkitt's lymphoma

GSE468	HC-G110	GEO	Available	13 primary medulloblastomas VS. 10 metastatic medulloblastomas

GSE4840	HG-U133A	GEO	Not available	3 samples from normal melanocyte culture VS. 12 samples from culture of cutaneous metastasis of melanoma
	
	HG-U133B	GEO	Not available	3 samples from normal melanocyte culture VS. 12 samples from culture of cutaneous metastasis of melanoma

GSE4843	HG-U133Plus2.0	GEO	Not available	45 samples from culture of cutaneous melanoma metastasis

GSE6369	HG-U133Plus2.0	GEO	Available	1 primary prostate carcinoma VS. 1 metastatic prostate carcinoma

GSE6919	HG-U95Av2	GEO	Available	65 primary prostate tumors VS. 25 metastatic prostate tumors
	
	HG-U95B	GEO	Available	66 primary prostate tumors VS. 25 metastatic prostate tumors
	
	HG-U95C	GEO	Available	65 primary prostate tumors VS. 25 metastatic prostate tumors

GSE7929	HG-U133A	GEO	Available	11 poorly metastatic melanoma VS. 21 highly metastatic melanoma

GSE7930	HG-U133A	GEO	Available	3 poorly metastatic prostate tumors VS. 3 highly metastatic prostate tumors

GSE7956	HG-U133A	GEO	Available	10 poorly metastatic melanoma VS. 29 highly metastatic melanoma

GSE8401	HG-U133A	GEO	Available	31 primary melanoma VS. 52 melanoma metastasis

The GEO or ArrayExpress accession numbers with the corresponding GeneChip model and the experimental conditions.

### Individual analyses

To process the datasets, we used alternative CDFs from AffyProbeMiner [23]. One CDF is needed per GeneChip model and three packages are needed per CDF. We used Version 1.8.0 of the "CDF distribution" packages. We used Version 1.0.0 of the "PROBE distribution" packages. We used Version 1.1.0 of the "Annotation distribution" packages. The CDFs used were "transcript-consistent", so each probe of a probe set maps to the same set of transcripts. We did not choose "gene-consistent" CDFs (probes of a probe set mapping to transcripts of the same set of genes) to avoid inconsistencies as recommended by Liu *et al*. [23]. The CDFs were chosen based on RefSeq and GeneBank information. The minimal size of a probe set was set to five probes as recommended by Liu *et al*. [23]. Pre-processing was performed with GCRMA [29] with the default parameters. Processing was performed with the Window Welch *t *test [35]. Due to a low number of conditions or of replicates to be statistically useful, datasets GSE4843 and GSE6369 could not be analyzed individually.

These individual analyses provided one gene list for each dataset or sub-dataset. For each gene list, we ranked the genes in ascending order of the p values of their differential expression, such that the most significantly over- or under- expressed genes are located at the top of the list.

### Intersections

The results from the individual analyses were grouped into 33 groups. For each group, the 50 most significant genes common to all datasets of the group were selected.

### Union intersections

The results from the individual analyses for the 17 metastasis datasets were grouped into 30 groups, while the results from the individual analyses for the 3 hypoxia datasets were grouped in one group. Each metastasis group was considered with the hypoxia group. For each couple of groups, the 50 most significant genes common to at least one dataset of the metastasis group and to at least one dataset of the hypoxia group were selected.

### Meta-analyses

The 22 datasets were merged into 14 meta-datasets. Alternative CDFs from AffyProbeMiner [23] were used. The meta-datasets were pre-processed with GCRMA [29] and processed with the Window Welch *t *test [35]. For each meta-dataset, the 50 most significant genes were selected.

### Visualization

Genes were thus selected by three approaches: intersections, union intersections and meta-analyses. Some were selected by two or three approaches. Those particular genes were submitted to the webtool DAVID (Database for Annotation, Visualization and Integrated Discovery) [36,37], version 6. The parameters of the "Functional Annotation Tool" were set to retrieve pathway maps from KEGG [38] and Biocarta [39]. And the parameters of the "Functional Annotation Clustering" (a part of the "Functional Annotation Tool") were set to the lowest level of stringency in order to obtain the largest number of maps.

### Computer and bioinformatic resources

Individual analyses, intersections, union intersections and meta-analyses were all run with the R statistical software [40] versions 2.4.0 and 2.6.0 and packages from Bioconductor [41] on a 64-bit computer with 4 gb of DDR (biprocessor dual-core Xeon 5160 3.0 Ghz, 8 × 500 gb RAID). Detailed scripts for every approach are provided as additional files (additional files 1, 2, 3, 4 and 5). However, brief descriptions for the individual analyses, intersections, union intersections and meta-analyses are provided here.

For each individual analysis, *expression sets *were obtained with the function justGCRMA (with default parameters) from the GCRMA [29] package. The *expression sets *were converted into a matrix with the function exprs, then split in two: condition A and condition B. P values were calculated with the Window Welch *t *test [35] with the *pegase *function from the Pegase package. Pegase is a package created by our laboratory that is not yet publicly available. Its function is to process microarray data after pre-processing. It requires an *expression set *as input and returns lists of p values for every well-known processing method as output. Here, *pegase *was run with default parameters. Fold changes were also calculated in the individual analyses.

A *data frame *was built for each dataset. The resulting *data frames *contained the AffyProbeMiner's probe set IDs for every probe set of the chip. They also contained the Entrez Gene IDs [42] corresponding to the probe sets as well as the p values and the fold changes. Since several Entrez Gene IDs can sometimes correspond to the same probe set, these particular probe sets as well as the corresponding p values and fold changes were repeated in the *data frames *with a different Entrez Gene ID each time.

For each *data frame*, the probe sets (and therefore the gene IDs and the fold changes) were ranked in ascending order of the p values of their differential expression. Intersections were created with the intersect function applied to the top lists of *data frames *previously described. For groups of datasets where only one GeneChip model was used, intersections were created at the probe set level. But for groups of datasets where several GeneChip models were used, intersections were created at the gene ID level.

Since the group of hypoxia datasets was built with two GeneChip models and was involved in every union intersection, union intersections were all created at the gene ID level. For each union intersection, gene IDs of the top lists of metastasis *data frames *were combined into a vector and gene IDs of the top lists of hypoxia *data frames *were combined into another one. Then, the intersect function was applied to those two vectors.

For meta-analyses, *expression sets *were obtained with the justGCRMA function (with default parameters). The *expression sets *were converted into a matrix with the exprs function, then split in two: condition A and condition B. P values were calculated with the Window Welch *t *test [35] (with default parameters) with the *pegase *function. The probe sets were then ranked in ascending order of the p values of their differential expression, and the 50 most significant ones were selected.

## Results and discussion

DNA microarrays and particularly Affymetrix GeneChips are widely used to measure the transcriptome of samples. Since the raw data can now be stored in numeric format, public databases have appeared and re-analysis of archived datasets has become common practice. Moreover, an increasing number of articles describe analyses combining several datasets. Specific methodologies for such meta-analyses are even published regularly [43-45]. While some of them are large-scale meta-analyses, others target more specific issues especially in the field of oncology. Here, we also provide a strategy for the meta-analysis of specific archived datasets, but the originality of this work is that it combines two different, but intimately related, biological processes: metastasis and hypoxia.

**Figure 1:**
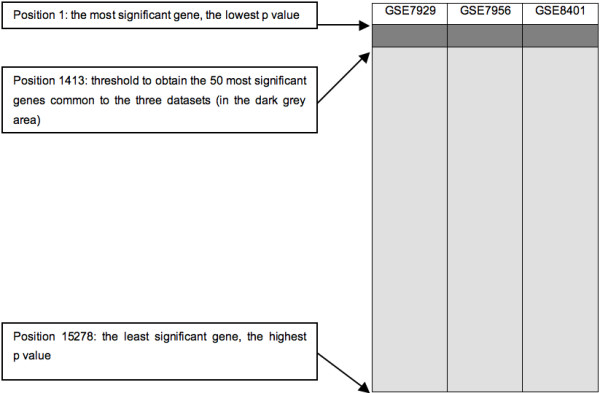
**Example of an intersection**. In each dataset, the probe sets were ranked in ascending order of the p values. The dark grey area is where the 50 most significant genes common to the three datasets of this particular intersection are found.

After individual analysis of the datasets retrieved from GEO [31] and ArrayExpress [32] (table 1), the results were combined in an approach called intersections. At each intersection, the 50 most significant genes common to several datasets were selected (figure 1). This arbitrary limit actually represents our upper limit for future *in vitro *validations. Like all statistical thresholds, this figure is arbitrary and is an attempted compromise. Indeed, a higher threshold would generate a number of genes that would be more difficult to interpret. Moreover, a higher threshold would lead to the selection of genes that are not statistically significant in the individual analyses. On the other hand, a lower threshold would allow for selection of a number of genes that would be easier to validate further and more statistically significant, but would also lead to a larger number of false negative genes. Thus, the threshold of 50 genes allows biological interpretation without selecting non significant genes. Since 33 different intersections were designed (Additional file 6), 1650 (33 × 50) different genes could potentially be selected. However, only 704 unique occurrences were obtained because some of them appeared in two or more lists. One advantage of this approach is that it uses the results from different GeneChip models. However, only few genes are common to all GeneChip models. For example, in this study, 8 GeneChip models were used but only 29 genes are represented on all 8 GeneChip models. This is due to some GeneChip models like HC-G110, HG-U133B, HG-U95B and HG-U95C in which few and/or poorly characterized genes are represented. So, intersections combining a large number of GeneChip models need to take a large number of genes into account to obtain the 50 most significant genes common to all the datasets considered.

A second approach combining the results from the individual analyses was the union intersections. By this approach, the 50 most significant genes common to at least one metastasis dataset and to at least one hypoxia dataset were selected at each union intersection. Thirty different union intersections were designed (Additional file 7), each combining a group of metastasis datasets and a group of hypoxia datasets. This approach ensures that every combination of results from the individual analyses takes the hypoxia datasets into account. This step was necessary since fewer hypoxia datasets than metastasis datasets were available. Moreover, union intersections do not require that a large number of genes be taken into account to obtain the 50 most significant genes as fewer are required for a gene to be selected. Out of the 1500 (30 × 50) possible genes, 269 unique occurrences were obtained.

**Figure 2:**
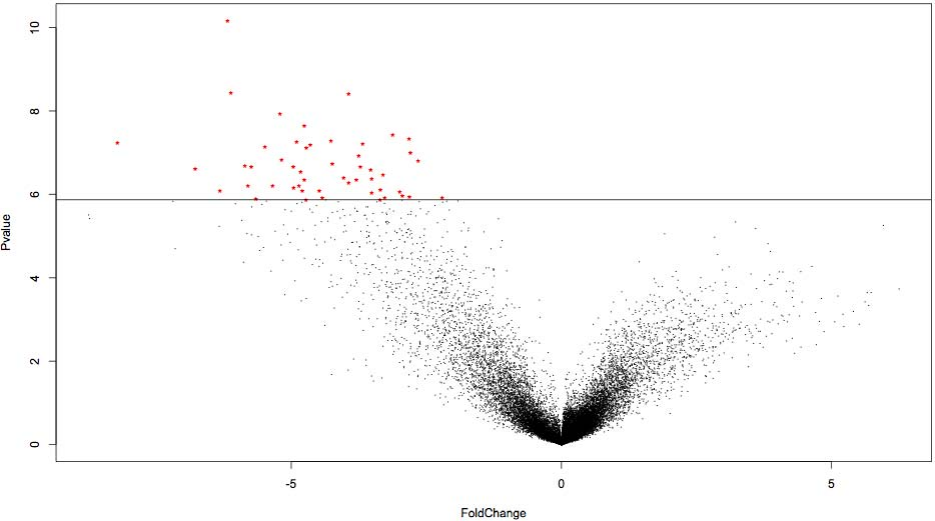
**Result of a meta-analysis**. The 50 most significant genes were selected in each volcano plot (log2 of the fold changes on the X axis and -log10 of the p values on the Y axis) resulting from the meta-analyses.

The last approach, called meta-analysis, was not based on the results from the individual analyses. Here, several datasets were merged into single datasets to artificially increase the number of replicates and thus increase the statistical power. Fourteen meta-datasets were designed (Additional file 8). A regular analysis was run on each meta-dataset and the 50 most significant genes were selected (figure 2). Since the meta-analyses were run from scratch, only datasets using the same GeneChip model could be combined. Again, a certain number of genes were present in more than one list: hence, meta-analyses provided 406 different genes out of the 700 (14 × 50) possible genes.

**Figure 3:**
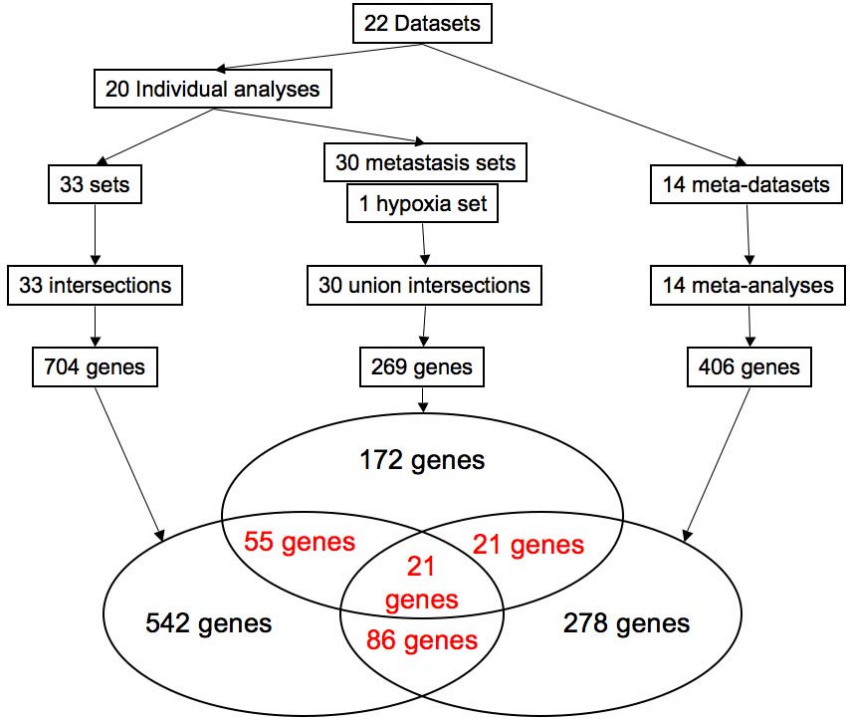
**Summary of the methodology**. The 22 datasets (or sub-datasets) were used to build several combinations in order to run intersections, union intersections and meta-analyses. These three approaches provided 704, 269 and 406 genes respectively. A Venn's diagram was then generated using this data.

**Figure 4:**
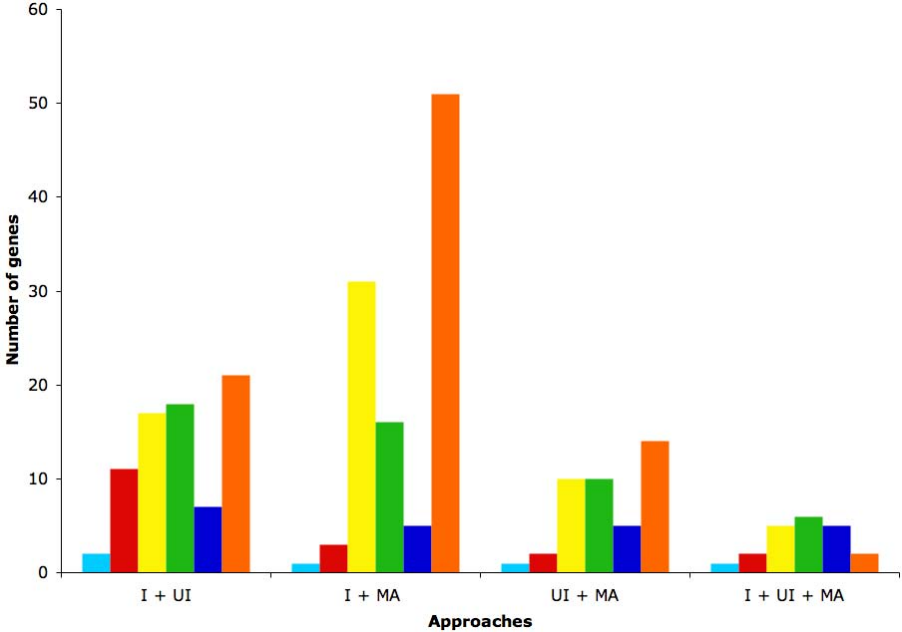
**Number of genes involved in processes of interest**. After the data mining in the literature, the 183 genes of interest were classified in several categories (light blue: known to be involved in hypoxia, red: known to be involved in cancer and hypoxia, yellow: known to be involved in cancer, green: known to be involved in cancer and metastasis, dark blue: known to be involved in cancer and metastasis and hypoxia, orange: not known to be involved in cancer or metastasis or hypoxia) in function of the combination of approaches (I for intersections, UI for union intersections and MA for meta-analyses).

The genes selected by these three approaches are represented in a Venn's diagram (figure 3). The 183 genes selected by more than one approach are considered as the genes of interest. They are listed in the Additional file 9. As six datasets or sub-datasets contain data obtained from melanomas and six others are from the prostate, two supplementary Venn's diagrams have been built based only on these datasets or sub-datasets (additional files 10 and 11). They highlight some genes within the 183 genes of interest. It is interesting to note that, even when half of the datasets are taken into account, so few genes are selected. This shows that the methodology is enriched by the number of datasets and thus by the diversity of information. Interestingly, 99 of the 183 genes of interest are described in the literature to be involved in cancer (figure 4, Additional file 9). For example, the methodology was able to find genes such as *JUNB*, *FOS *and *ATF3*, all members of the AP-1 complex. AP-1 is a transcription factor involved in cell proliferation and differentiation. It has often been described as a "double-edged sword" since its effect can be the repression as well as the promotion of tumorigenesis [46]. Indeed, AP-1 transcription factors are dimers composed of the JUN, FOS and ATF protein families. Depending on the exact AP-1 composition, it promotes or represses tumorigenesis. JUNB acts as a repressor of cell proliferation through its repression activity on the cyclin D1, an essential element in the cell cycle [47,48]. On the contrary, FOS and ATF3 induce oncogenic transformation [49]. Another well-described gene in cancer selected by the methodology is *TP63*. TP63 is a transcription factor sharing a large degree of homology with TP53. It is involved in the development of stratified epithelial tissues [50]. *TP63 *has two different promoters and is the target of alternative splicing events leading to the existence of several isoforms. For example, *TAp63 *is a tumor suppressor while Δ*Np63 *is an oncogene since it antagonizes *TAp63 *[51].

Of the 99 genes known to be involved in cancer, 39 have been described to regulate metastasis (figure 4, Additional file 9). For example, the gene *SERPINE1*, coding for plasminogen activator inhibitor 1 (PAI-1), was selected by the methodology. *SERPINE1 *plays a central role in several key steps of metastasis. First, it is able to catalyse degradation of the extracellular matrix to allow penetration of metastatic cancer cells into tissues. Second, when PAI-1 is bound to the plasminogen activator, it is able to modulate cell adhesion by decreasing its affinity for vitronectin and increasing its affinity for endocytic receptors, thus enabling cell migration. Moreover, *SERPINE1 *enhances cell proliferation [52]. Another example of a gene selected by the methodology and well described as involved in the metastatic process is the gene coding for matrix metalloproteinase 7 (MMP7). Matrix metalloproteinases are enzymes that cleave the extracellular matrix in normal processes such as morphogenesis, angiogenesis and tissue repair. It was also often described in recent years to be involved in cancer processes such as tumorigenesis, invasion and metastasis [53-55].

Lastly, 21 genes of the 183 selected by the methodology are linked to hypoxia (figure 4, additional file 9). *VEGFA *is probably the best example of such a gene selected by the methodology. *VEGFA *has been largely described to act on endothelial cells to promote the development of vasculature in embryos. Moreover, through the transcription factor HIF-1, hypoxia induces the production of VEGFA to stimulate angiogenesis in newly-formed organs. The same mechanisms are triggered during tumor growth. Indeed, when the tumor size increases, it becomes hypoxic, thus leading to the stabilization of HIF-1 that promotes the transcription of *VEGFA*. VEGFA then stimulates angiogenesis in the tumor [56,57]. *ID2 *is another example of genes selected by the methodology and known to be responsive to hypoxia. ID2 belongs to the family of ID proteins which are transcriptional regulators that inhibit basic helix-loop-helix transcription factors in processes such as proliferation, differentiation, development and angiogenesis. It is interesting to note that ID2 is able to inhibit VEGFA and thus limit metastasis [58]. Surprisingly, however, *ID2 *is a target of HIF-1 since there are two HIF-1 binding sites within *ID2 *gene regulatory sequences. Besides, studies have shown that *ID2 *expression is induced under hypoxic conditions [59].

Another strong argument in favor of the proposed methodology is the ability of DAVID [36,37] to classify 179 of the 183 genes in 24 different pathways. It is noteworthy that 8 of these pathways are clearly involved in cancer (table 2). For example, "glioma" was one of the pathways retrieved by DAVID [36,37]. Gliomas are cancer which initiate with the oncogenic transformation of a brain or spinal cord cell. There are several types of gliomas which vary in the type of cell transformed. The most common types are those which affect ependymal cells, astrocytes and oligodendrocytes [60]. Another example is "prostate cancer". Prostate cancer is one of the most frequent types of cancer in men. It often develops in patients over the age of 50. This type of cancer is subject to metastasis, particularly in the bones and lymph nodes [61]. "Colorectal cancer" was also retrieved by DAVID [36,37]. This type of cancer is also one of the most common and one of the main cancer-related cause of death. Oncogenic transformation occurs in the adenomatous polyps in the colon and cancer cells can metastasize to the liver, principally [62].

**Table 2:** DAVID information

	Pathways	Databases	Genes
Cancer	Prostate cancer	KEGG	MAPK1, IGF1, MAP2K1, CCNE2, NFKBIA
	
	Chronic myeloid leukemia	KEGG	MAPK1, MAP2K1, NFKBIA
	
	Colorectal cancer	KEGG	FOS, MAPK1, MAP2K1
	
	Renal cell carcinoma	KEGG	VEGFA, MAPK1, MAP2K1, PAK6
	
	Pancreatic cancer	KEGG	STAT1, VEGFA, MAPK1, MAP2K1
	
	Bladder cancer	KEGG	VEGFA, MAPK1, MAP2K1
	
	Glioma	KEGG	MAPK1, IGF1, MAP2K1
	
	Melanoma	KEGG	MAPK1, IGF1, MAP2K1

Proliferation and cell motility	Focal adhesion	KEGG	FLNC, VEGFA, MAPK1, SPP1, IGF1, MAP2K1, PAK6, LAMA3, MYL9
	
	MAPK signalling pathway	KEGG, BIOCARTA	FLNC, NR4A1, FOS, MAPK1, DUSP1, MAP2K1, DUSP8, NFKBIA
	
	VEGF signalling pathway	KEGG	VEGFA, HSPB1, MAPK1, MAP2K1
	
	ErbB signalling pathway	KEGG	MAPK1, MAP2K1, PAK6, ERBB3
	
	Regulation of actin cytoskeleton	KEGG	ACTG2, MAPK1, MAP2K1, ACTC1, PAK6, MYL9

Pathogen recognition and phagocytosis	Pathogenic Escherichia coli infection - EPEC	KEGG	YWHAZ, TUBB2B, TUBB2A, TUBB2C, TUBB4
	
	Pathogenic Escherichia coli infection - EHEC	KEGG	YWHAZ, TUBB2B, TUBB2A, TUBB2C, TUBB4
	
	T Cell Receptor Signalling Pathway	BIOCARTA	FOS, MAP2K1, NFKBIA
	
	Toll-like receptor signalling pathway	KEGG	STAT1, FOS, MAPK1, SPP1, MAP2K1, NFKBIA
	
	fMLP induced chemokine gene expression in HMC-1 cells	BIOCARTA	MAPK1, MAP2K1, NFKBIA
	
	Fc Epsilon Receptor I Signalling in Mast Cells	BIOCARTA	FOS, MAPK1, MAP2K1

Other	Keratinocyte Differentiation	BIOCARTA	MAPK1, MAP2K1, NFKBIA
	
	Gap junction	KEGG	TUBB2B, MAPK1, MAP2K1, TUBB2A, TUBB2C, TUBB4
	
	NFAT and Hypertrophy of the heart	BIOCARTA	MAPK1, IGF1, MAP2K1
	
	Long-term depression	KEGG	MAPK1, IGF1, MAP2K1
	
	Cadmium induces DNA synthesis and proliferation in macrophages	BIOCARTA	FOS, MAPK1, MAP2K1, NFKBIA

DAVID classified 179 of the 183 genes of interest into 24 pathways from KEGG or Biocarta. Column 3 presents the genes involved in each specific pathway.

Five other pathways retrieved by DAVID [36,37] are related to proliferation and cell motility (table 2): "focal adhesion", "MAPK signalling pathway", "VEGF signalling pathway", "ErbB signalling pathway" and "regulation of actin cytoskeleton". The focal adhesions are macromolecular structures at the contact points between the cell and the extracellular matrix [63]. They enable tissue remodelling, cell migration and embryogenesis through regulation of the structure of the cytoskeleton, cell adhesion sites and membrane protrusions [64]. The mitogen-activated protein kinase (MAPK) signalling pathway is a cascade involved in the regulation of cellular processes such as cell proliferation, differentiation and stress response [65]. This regulation occurs through the phosphorylation of key proteins in these processes [66]. The VEGF signalling pathway is activated to ensure proliferation and migration of endothelial cells during normal processes such as vasculogenesis as well as pathological processes such as tumor growth [67]. The ErbB signalling pathway is actually composed of several transmembrane receptors able to trigger several signalling pathways when they bind to an extracellular growth factor molecule. These signalling pathways themselves regulate biological processes such as proliferation, differentiation, cell motility and survival [68,69]. The regulation of actin cytoskeleton includes mechanisms which allow for the functions of microfilaments. Microfilaments are responsible for cell shape, intracellular transport and cell motility [70].

**Figure 5:**
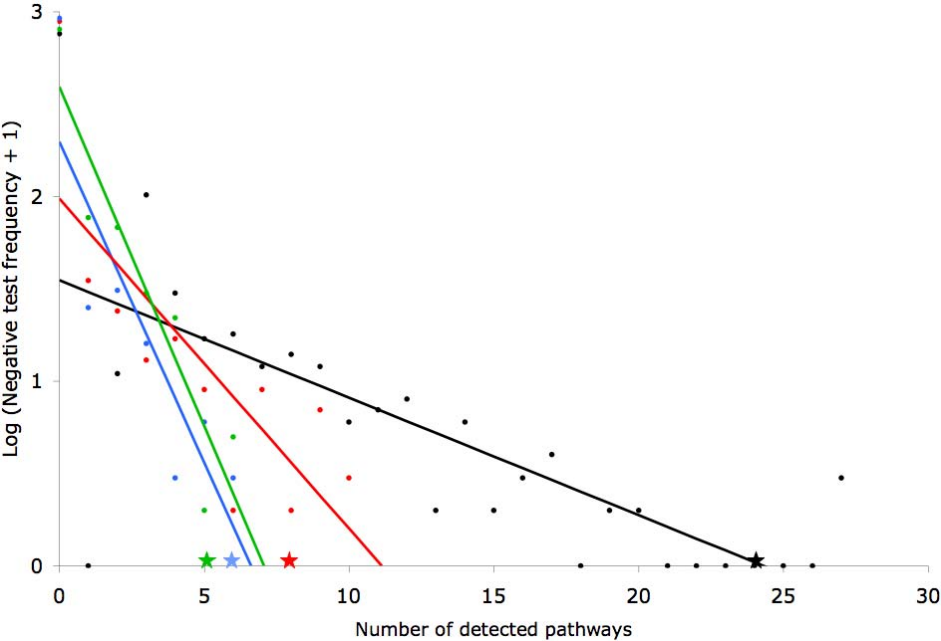
**Number of pathways detected by DAVID in negative controls**. 1000 lists of 183 random genes were submitted to DAVID. The number of pathways detected per test is presented on the X axis and the logarithm of the frequency of the tests (+ 1 to avoid log (0)) is presented on the Y axis. The black dots show the total number of pathways detected per test and the black star indicates the total number of pathways detected with the 183 genes of interest selected by the methodology. The red dots show the number of pathways directly involved in cancer detected per test and the red star indicates the number of pathways directly involved in cancer detected with the 183 genes of interest selected by the methodology. The green dots show the number of pathways involved in proliferation and cell motility detected per test and the green star indicates the number of pathways involved in proliferation and cell motility detected with the 183 genes of interest selected by the methodology. Lastly, the blue dots show the number of pathways involved in pathogen recognition and phagocytosis detected per test and the blue star indicates the number of pathways involved in pathogen recognition and phagocytosis detected with the 183 genes of interest selected by the methodology.

As a first negative control, 183 genes were randomly selected. Only 62 of those random genes were found in the literature to be involved in cancer, among which 11 are described to regulate metastasis and 8 are linked to hypoxia. And as a second negative control, 1000 selections of 183 random genes were run. These 1000 lists of random genes were submitted to DAVID to see how many pathways would be highlighted by chance. This was done for the total number of pathways, for the number of pathways directly involved in cancer and for the number of pathways involved in proliferation and cell motility (figure 5). For the total number of pathways, only two tests gave better results than the 183 genes of interest selected by the methodology. For the number of pathways directly involved in cancer, only nine tests gave equal or better results than the 183 genes of interest selected by the methodology. Lastly, for the number of pathways involved in proliferation and cell motility, only five tests gave equal or better results than the 183 genes of interest selected by the methodology. This indicates that the probability of obtaining the results observed with the 183 genes of interest by chance is between 0,01 and 0,001. Taken together, these results indicate that the methodology is able to find genes actually involved in a particular biological process or even genes involved in a combination of processes (here metastasis and hypoxia).

Since the data on the involvement of the genes of interest in cancer, metastasis and hypoxia support the methodology, we propose that the 84 genes (183 - 99) not known to be involved in cancer to be good candidates for involvement in development of the cancer and in particular in metastasis induced by hypoxia. Obviously, further analyses are required. However, it is already interesting to note that 6 out of the 24 pathways retrieved by DAVID [36,37] concern pathogen recognition and phagocytosis (table 2): "pathogenic *Escherichia coli *infection - EPEC", "pathogenic *Escherichia coli *infection - EHEC", "toll-like receptor signalling pathway", "fMLP induced chemokine gene expression in HMC-1 cells", "Fc epsilon receptor I signalling in mast cells" and "T cell receptor signalling pathway". Thus, we decided to further examine the genes in these pathways and their involvement in cancer, metastasis and hypoxia.

Enteropathogenic *Escherichia coli *(EPEC) and enterohemorrhagic *Escherichia coli *(EHEC) are two pathogens characterized by their ability to cause attaching and effacing lesions. This ability is mainly encoded by the locus of the enterocyte effacement pathogenicity island which includes four genes: *Tir*, *Map*, *EspF *and *EspG*. Interestingly, *Tir *codes for a protein that allows for the accretion of actin [71]. This pathway is thus related to regulation of the actin cytoskeleton pathway identified by the methodology. These three pathways ("pathogenic *Escherichia coli *infection - EPEC", "pathogenic *Escherichia coli *infection - EHEC" and "regulation of actin cytoskeleton") could be activated in metastasis to enable rearrangement of the cytoskeleton and migration of the cell.

The toll-like receptor signalling pathway is composed of a set of receptors able to recognize specific molecules from pathogens. This recognition results in an innate immune response by the activation of inflammatory genes. However, every receptor is specific to a particular signal and triggers a specific cellular response, so the functions of the different toll-like receptors are not redundant [72]. "fMLP-induced chemokine gene expression in HMC-1 cells" is a pathway activated in neutrophils when a bacterial infection occurs. This pathway activates NADPH oxidase that produces reactive oxygen species to kill the bacteria. It also activates genes coding for chemokines to attract other innate immune cells to fight the infection [73]. "Fc Epsilon Receptor I Signalling in Mast Cells" is a defence pathway against some parasites. When activated, mast cells can trigger inflammation [74]. The "T Cell Receptor Signalling Pathway" is a pathway activated when a T Cell Receptor binds to a peptide from a foreign organism. This event activates T cells and immunity [39].

Surprisingly, these pathways have no link with cancer, metastasis or hypoxia, but were identified by our methodology. Moreover, the second negative control assessed the number of pathways involved in pathogen recognition and phagocytosis and only two tests over 1000 trials gave results equal to those with the 183 genes of interest selected by the methodology (figure 5). *In vitro *confirmation of their expression in cancer cell lines with high potential would confirm the relevance of the methodology we propose and the involvement of these genes and pathways in the metastasis of cancer cells. Moreover, functional analysis of the products of these genes should provide new keys to the understanding of the mechanisms involved in the developement of metastases.

## Conclusion

We describe a methodology able to identify new genes involved in specific conditions from several microarray datasets. This statement is supported by the fact that this methodology was able to identify genes already known to be involved in the biological processes which we studied. The next step will be *in silico *validation by analysing the expression profile of our genes of interest in publicly available expression profile datasets from different cancer cell lines and *in vitro *validation by qRT-PCR.

The first to be investigated are the genes involved in pathogen recognition and phagocytosis. Indeed, several elements indicate that these pathways may be involved in cancer and particularly in the metastatic process induced by hypoxia. Not only the genes selected by the methodology will be tested. We actually plan to test close neighbouring genes inside the pathways in order to validate large portions of pathways or entire pathways instead of single genes.

This is likely to improve our understanding of the mechanisms underlying this pathology and provide new opportunities to fight it.

## Competing interests

The authors declare that they have no competing interests.

## Authors' contributions

MP carried out all the experiments, AG and FB participated in the development of the R scripts, BDM participated in the retrieval and the analysis of the informations from DAVID, BDH and EB participated in the selection and the retrieval of the datasets from GEO and ArrayExpress, CM and ED conceived the study, participated in its design and helped to draft the manuscript. All authors read and approved the final manuscript.

## Pre-publication history

The pre-publication history for this paper can be accessed here:

http://www.biomedcentral.com/1471-2407/10/176/prepub

## Supplementary Material

Additional file 1 **R script for individual analyses**. The HG-U133A Affymetrix GeneChip was used in this example of script. The script is in R language. Some objects and values, symbolyzed here by X or Y, have to be replace according to the dataset analyzed. CDF packages can vary according to the GeneChip model analyzed. Click here for file

Additional file 2 **R script for data frames**. The HG-U133A Affymetrix GeneChip was used in this example of script. The script is in R language. Some objects and values like the length of some vectors have to be replace according to the GeneChip model analyzed. CDF packages can vary according to the GeneChip model analyzed. Click here for file

Additional file 3 **R script for intersections**. The script is in R language. Some objects and values, symbolyzed here by X have to be replace according to the datasets involved in the intersection. Click here for file

Additional file 4 **R script for union intersections**. The script is in R language. Some objects and values, symbolyzed here by X have to be replace according to the datasets involved in the union intersection. Click here for file

Additional file 5 **R script for meta-analyses**. The HG-U133A Affymetrix GeneChip was used in this example of script. The script is in R language. Some objects and values, symbolyzed here by X or Y, have to be replace according to the meta-dataset analyzed. CDF packages can vary according to the GeneChip model analyzed. Click here for file

Additional file 6 **Intersections**. 33 groups of datasets were designed based on the experimental conditions and/or the GeneChip model. Click here for file

Additional file 7 **Union intersections**. 30 groups of metastasis datasets were designed based on the experimental conditions and/or the GeneChip model. All were compared to the group of hypoxia datasets. Click here for file

Additional file 8 **Meta-datasets**. 14 meta-datasets were designed based on the experimental conditions. Click here for file

Additional file 9 **Table of references**. This table reports the number of the references in the references section for all 183 genes of interest. These are the publications where those genes were shown to be involved in cancer (column 2), in metastasis (column 3) and/or in hypoxia (column 4). Click here for file

Additional file 10 **Venn's diagram for the prostate datasets**. The 6 prostate specific datasets (or sub-datasets) were used to run two intersections, two union intersections and one meta-analysis. These three approaches provided 87, 74 and 48 genes respectively. A Venn's diagram was then generated using these data. Click here for file

Additional file 11 **Venn's diagram for the melanoma datasets**. The 6 melanoma specific datasets (or sub-datasets) were used to run three intersections, three union intersections and three meta-analyses. These three approaches provided 144, 97 and 63 genes respectively. A Venn's diagram was then generated using these data. Click here for file
