# Supplementary material for: Meta-analysis of archived DNA microarrays identifies genes regulated by hypoxia and involved in a metastatic phenotype in cancer cells
Source: BMC Cancer. 2010 Apr 30;10:176. doi: 10.1186/1471-2407-10-176 (PMC2880990; doi:10.1186/1471-2407-10-176)
Supplement: Additional file 2 — R script for data frames. The HG-U133A Affymetrix GeneChip was used in this example of script. The script is in R language. Some objects and values like the length of some vectors have to be replace according to the GeneChip model analyzed. CDF packages can vary according to the GeneChip model analyzed. [file 1471-2407-10-176-S2.PDF]

```
load("/…/…") # to load the object containing the list of p
values of a dataset
library(hgu133atranscriptccds)
data(hgu133atranscriptccds)
library(hgu133atranscriptccdscdf) # these three commands allow
to load the AffyProbeMiner's CDF and the related informations
a<-
mget(ls(env=hgu133atranscriptccdscdf),hgu133atranscriptccdsENT
REZGENEID) # to get the list of IDs from the Entrez database
for every probe set of the AffyProbeMiner's CDF
b<-c()
for(i in 1:15278){
b[i]<-a[[i]][1]}
d<-c()
for(i in 1:15278){
d[i]<-a[[i]][2]}
e<-which(d>0)
d<-d[!is.na(d)]
f<-c()
for(i in 1:15278){
f[i]<-a[[i]][3]}
g<-which(f>0)
f<-f[!is.na(f)]
h<-c()
for(i in 1:15278){
h[i]<-a[[i]][4]}
j<-which(h>0)
h<-h[!is.na(h)]
k<-c()
for(i in 1:15278){
k[i]<-a[[i]][5]}
l<-which(k>0)
k<-k[!is.na(k)]
m<-c()
for(i in 1:15278){
m[i]<-a[[i]][6]}
n<-which(m>0)
m<-m[!is.na(m)]
o<-c()
for(i in 1:15278){
o[i]<-a[[i]][7]}
p<-which(o>0)
o<-o[!is.na(o)]
q<-c()
for(i in 1:15278){
q[i]<-a[[i]][8]}
r<-which(q>0) # these commands allow to get the indices of the
probe sets corresponding to several IDs
q<-q[!is.na(q)] # these loops allow to create vectors
containing every ID of Entrez for each probe set since some
probe sets correspond to several IDs
```

```
geneID<-c(b,d,f,h,k,m,o,q) # to create a vector containing all
the IDs for all the probe sets
a<-names(PV) # to get the names of the probe sets. PV is the
object loaded with the first command
b<-a[e]
d<-a[g]
f<-a[j]
h<-a[l]
k<-a[n]
m<-a[p]
o<-a[r] # these commands allow to get the names of the probe
sets corresponding to several IDs
probeset<-c(a,b,d,f,h,k,m,o) # to create a vector containing
all the names of all the probe sets
a<-PV
b<-a[e]
d<-a[g]
f<-a[j]
h<-a[l]
k<-a[n]
m<-a[p]
o<-a[r] # these commands allow to get the p values of the
probe sets corresponding to several IDs
PValue<-c(a,b,d,f,h,k,m,o) # to create a vector containing all
the p values of all the probe sets
DF<-data.frame(probeset,geneID,PValue) # to create a data
frame containing for every probe set the corresponding IDs and
the corresponding p value
```
