# Supplementary material for: Meta-analysis of archived DNA microarrays identifies genes regulated by hypoxia and involved in a metastatic phenotype in cancer cells
Source: BMC Cancer. 2010 Apr 30;10:176. doi: 10.1186/1471-2407-10-176 (PMC2880990; doi:10.1186/1471-2407-10-176)
Supplement: Additional file 3 — R script for intersections. The script is in R language. Some objects and values, symbolyzed here by X have to be replace according to the datasets involved in the intersection. [file 1471-2407-10-176-S3.PDF]

```
load("/…/DF1")
load("/…/DF2")
load("/…/DF3") # to load the data frames of the datasets
intervening in the intersection
a<-DF1[order(DF1$PValue),]
b<-DF2[order(DF2$PValue),]
d<-DF3[order(DF3$PValue),] # to rank the probe sets in
ascending order of their p values
e<-a$geneID[1:X]
f<-b$geneID[1:X]
g<-d$geneID[1:X] # to select the necessary number (X) of genes
to get 50 common genes to the three top lists of p values
a<-intersect(e,f)
b<-intersect(g,a) # to select the 50 common genes
```
