# Supplementary material for: Meta-analysis of archived DNA microarrays identifies genes regulated by hypoxia and involved in a metastatic phenotype in cancer cells
Source: BMC Cancer. 2010 Apr 30;10:176. doi: 10.1186/1471-2407-10-176 (PMC2880990; doi:10.1186/1471-2407-10-176)
Supplement: Additional file 4 — R script for union intersections. The script is in R language. Some objects and values, symbolyzed here by X have to be replace according to the datasets involved in the union intersection. [file 1471-2407-10-176-S4.PDF]

```
load("/…/DF1")
load("/…/DF2")
load("/…/DF3") # to load the data frames of the metastasis
datasets intervening in the union intersection
load("/…/DF4")
load("/…/DF5")
load("/…/DF6") # to load the data frames of the hypoxia
datasets intervening in the union intersection
Z1<-DF1[order(DF1$PValue),]
Z2<-DF2[order(DF2$PValue),]
Z3<-DF3[order(DF3$PValue),]
Z4<-DF4[order(DF4$PValue),]
Z5<-DF5[order(DF5$PValue),]
Z6<-DF6[order(DF6$PValue),] # to rank the probe sets in
ascending order of their p values
a<-Z1$geneID[1:X]
a<-as.character(a)
b<-Z2$geneID[1:X]
b<-as.character(b)
d<-Z3$geneID[1:X]
d<-as.character(d)
e<-c(a,b,d)
A<-Z4$geneID[1:X]
A<-as.character(A)
B<-Z5$geneID[1:X]
B<-as.character(B)
D<-Z6$geneID[1:X]
D<-as.character(D)
E<-c(A,B,D) # to select the necessary number (X) of genes to
get 50 common genes to at least one top list of p values of
the metastasis datasets and to at least one top list of p
values of the hypoxia datasets
union<-intersect(e,E) # to select the 50 common genes
```
