# Supplementary material for: Meta-analysis of archived DNA microarrays identifies genes regulated by hypoxia and involved in a metastatic phenotype in cancer cells
Source: BMC Cancer. 2010 Apr 30;10:176. doi: 10.1186/1471-2407-10-176 (PMC2880990; doi:10.1186/1471-2407-10-176)
Supplement: Additional file 5 — R script for meta-analyses. The HG-U133A Affymetrix GeneChip was used in this example of script. The script is in R language. Some objects and values, symbolyzed here by X or Y, have to be replace according to the meta-dataset analyzed. CDF packages can vary according to the GeneChip model analyzed. [file 1471-2407-10-176-S5.PDF]

```
setwd("/…/…") # to load the dataset in the environment
library(hgu133acdf) # to load the Affymetrix CDF in the
environment
library(gcrma) # to load the GCRMA package in the environment
library(pegase) # to load the Pegase package in the
environment
library(hgu133atranscriptccds)
data(hgu133atranscriptccds) # this couple of commands allows
to load the AffyProbeMiner's CDF
hgu133atranscriptccdsdim<-hgu133adim # to replace the
Affymetrix CDF by the AffyProbeMiner's CDF
a<-justGCRMA(cdfname="hgu133atranscriptccds") # to use the
AffyProbeMiner's CDF and to pre-process the data into an
expression set
b<-exprs(a) # to convert the expression set into a matrix
d<-b[,(1:X)]
e<-b[,(X+1:Y)] # these two commands allow to separate the
matrix between the two conditions of the experiment
f<-
pegase(A=d,B=e,steps=c("prepare","run"),methods=c("win.welch")
) # to process the data, the two first arguments specify the
matrix of each condition, the third argument specifies the
steps of the processing, the last argument specifies the
method used, here the Window Welch t test. The p values are
stored in f$pvals
g<-sort(f$pvals) # to rank the probe sets in ascending order
of their p values
h<-g[(1:50)] # to select the 50 most significant probe sets
```
