# Supplementary material for: Meta-analysis of archived DNA microarrays identifies genes regulated by hypoxia and involved in a metastatic phenotype in cancer cells
Source: BMC Cancer. 2010 Apr 30;10:176. doi: 10.1186/1471-2407-10-176 (PMC2880990; doi:10.1186/1471-2407-10-176)
Supplement: Additional file 7 — Union intersections. 30 groups of metastasis datasets were designed based on the experimental conditions and/or the GeneChip model. All were compared to the group of hypoxia datasets. [file 1471-2407-10-176-S7.PDF]

	Experimental conditions	GeneChip models	Datasets
**Metastasis group 1**	Primary tumor VS. metastasis	HG-U133A	E-GEOD-1323, E-GEOD-2280, GSE2280, GSE2603, GSE7929, GSE7956, GSE8401
**Metastasis group 2**	Primary tumor VS. metastasis, normal tissue VS. Metastasis	HG-U133A	E-GEOD-1323, E-GEOD-2280, GSE2280, GSE2603, GSE4840 (HG-U133A), GSE7929, GSE7956, GSE8401
**Metastasis group 3**	Primary tumor VS. metastasis, poorly metastatic tissue VS. highly metastatic tissue	HG-U133A	E-GEOD-1323, E-GEOD-2280, GSE2280, GSE2603, GSE7929, GSE7930, GSE7956, GSE8401
**Metastasis group 4**	Primary tumor VS. metastasis, normal tissue VS. metastasis, poorly metastatic tissue VS. highly metastatic tissue	HG-U133A	E-GEOD-1323, E-GEOD-2280, GSE2280, GSE2603, GSE4840 (HG-U133A), GSE7929, GSE7930, GSE7956, GSE8401
**Metastasis group 5**	Primary tumor VS. metastasis	HG-U95Av2	E-MEXP-44 (HG-U95Av2), GSE6919 (HG-U95Av2)
**Metastasis group 6**	Primary tumor VS. ùetastasis	HG-U133A, HG-U95Av2	E-GEOD-1323, E-GEOD-2280, E-MEXP-44 (HG-U95Av2), GSE2280, GSE2603, GSE6919 (HG-U95Av2), GSE7929, GSE7956, GSE8401
**Metastasis group 7**	Primary tumor VS. metastasis, normal tissue VS. Metastasis	HG-U133A, HG-U95Av2	E-GEOD-1323, E-GEOD-2280, E-MEXP-44 (HG-U95Av2), GSE2280, GSE2603, GSE4840 (HG-U133A), GSE6919 (HG-U95Av2), GSE7929, GSE7956, GSE8401
**Metastasis group 8**	Primary tumor VS. metastasis, poorly metastatic tissue VS. highly metastatic tissue	HG-U133A, HG-U95Av2	E-GEOD-1323, E-GEOD-2280, E-MEXP-44 (HG-U95Av2), GSE2280, GSE2603, GSE6919 (HG-U95Av2), GSE7929, GSE7930, GSE7956, GSE8401
**Metastasis group 9**	Primary tumor VS. metastasis, normal tissue VS. metastasis, poorly metastatic tissue VS. highly metastatic tissue	HG-U133A, HG-U95Av2	E-GEOD-1323, E-GEOD-2280, E-MEXP-44 (HG-U95Av2), GSE2280, GSE2603, GSE4840 (HG-U133A), GSE6919 (HG-U95Av2), GSE7929, GSE7930, GSE7956, GSE8401
**Metastasis group 10**	Primary tumor VS. metastasis	HG-U133A, HG-U133Plus2.0	E-GEOD-1323, E-GEOD-2280, GSE2280, GSE2603, GSE3325, GSE7929, GSE7956, GSE8401
**Metastasis group 11**	Primary tumor VS. metastasis, normal tissue VS. Metastasis	HG-U133A, HG-U133Plus2.0	E-GEOD-1323, E-GEOD-2280, GSE2280, GSE2603, GSE3325, GSE4840 (HG-U133A), GSE7929, GSE7956, GSE8401
**Metastasis group 12**	Primary tumor VS. metastasis, poorly metastatic tissue VS. highly metastatic tissue	HG-U133A, HG-U133Plus2.0	E-GEOD-1323, E-GEOD-2280, GSE2280, GSE2603, GSE3325, GSE7929, GSE7930, GSE7956, GSE8401
**Metastasis group 13**	Primary tumor VS. metastasis, normal tissue VS. metastasis, poorly metastatic tissue VS. highly metastatic tissue	HG-U133A, HG-U133Plus2.0	E-GEOD-1323, E-GEOD-2280, GSE2280, GSE2603, GSE3325, GSE4840 (HG-U133A), GSE7929, GSE7930, GSE7956, GSE8401
**Metastasis group 14**	Primary tumor VS. metastasis	HG-U133Plus2.0, HG-U95Av2	E-MEXP-44 (HG-U95Av2), GSE3325, GSE6919 (HG-U95Av2)
**Metastasis group 15**	Primary tumor VS. metastasis	HG-U133A, HG-U133Plus2.0, HG-U95Av2	E-GEOD-1323, E-GEOD-2280, E-MEXP-44 (HG-U95Av2), GSE2280, GSE2603, GSE3325, GSE6919 (HG-U95Av2), GSE7929, GSE7956, GSE8401
**Metastasis group 16**	Primary tumor VS. metastasis, normal tissue VS. Metastasis	HG-U133A, HG-U133Plus2.0, HG-U95Av2	E-GEOD-1323, E-GEOD-2280, E-MEXP-44 (HG-U95Av2), GSE2280, GSE2603, GSE3325, GSE4840 (HG-U133A), GSE6919 (HG-U95Av2), GSE7929, GSE7956, GSE8401
**Metastasis group 17**	Primary tumor VS. metastasis, poorly metastatic tissue VS. highly metastatic tissue	HG-U133A, HG-U133Plus2.0, HG-U95Av2	E-GEOD-1323, E-GEOD-2280, E-MEXP-44 (HG-U95Av2), GSE2280, GSE2603, GSE3325, GSE6919 (HG-U95Av2), GSE7929, GSE7930, GSE7956, GSE8401
**Metastasis group 18**	Primary tumor VS. metastasis, normal tissue VS. metastasis, poorly metastatic tissue VS. highly metastatic tissue	HG-U133A, HG-U133Plus2.0, HG-U95Av2	E-GEOD-1323, E-GEOD-2280, E-MEXP-44 (HG-U95Av2), GSE2280, GSE2603, GSE3325, GSE4840 (HG-U133A), GSE6919 (HG-U95Av2), GSE7929, GSE7930, GSE7956, GSE8401

**Metastasis group 19**	Primary tumor VS. metastasis, normal tissue VS. Metastasis	HG-U133B, HG-U95B	GSE4840 (HG-U133B), GSE6919 (HG-U95B)
**Metastasis group 20**	Primary tumor VS. metastasis	HG-U133A, HG-U133Plus2.0, HG-U95Av2, HG-U95B, HG-U95C, HU-geneFL, HC-G110	E-GEOD-1323, E-GEOD-2280, E-MEXP-44, GSE2280, GSE2603, GSE3325, GSE468, GSE6919, GSE7929, GSE7956, GSE8401
**Metastasis group 21**	Primary tumor VS. metastasis, normal tissue VS. Metastasis	All GeneChip models	E-GEOD-1323, E-GEOD-2280, E-MEXP-44, GSE2280, GSE2603, GSE3325, GSE468, GSE4840, GSE6919, GSE7929, GSE7956, GSE8401
**Metastasis group 22**	Primary tumor VS. metastasis, poorly metastatic tissue VS. highly metastatic tissue	HG-U133A, HG-U133Plus2.0, HG-U95Av2, HG-U95B, HG-U95C, HU-geneFL, HC-G110	E-GEOD-1323, E-GEOD-2280, E-MEXP-44, GSE2280, GSE2603, GSE3325, GSE468, GSE6919, GSE7929, GSE7930, GSE7956, GSE8401
**Metastasis group 23**	Primary tumor VS. metastasis, normal tissue VS. metastasis, poorly metastatic tissue VS. highly metastatic tissue	All GeneChip models	E-GEOD-1323, E-GEOD-2280, E-MEXP-44, GSE2280, GSE2603, GSE3325, GSE468, GSE4840, GSE6919, GSE7929, GSE7930, GSE7956, GSE8401
**Metastasis group 24**	Squamous cell carcinoma of the oral cavity VS. corresponding lymph node metastases	HG-U133A	E-GEOD-2280, GSE2280
**Metastasis group 25**	Head and neck squamous cell carcinoma VS. corresponding lymph node metastases	HG-U95Av2, HU-geneFL	E-MEXP-44
**Metastasis group 26**	Primary prostate cancer VS. metastases	HG-U133Plus2.0, HG-U95B, HG-U95C	GSE3325, GSE6919 (HG-U95B, HG-U95C)
**Metastasis group 27**	Primary prostate cancer VS. metastases, poorly metastatic prostate tumors VS. highly metastatic prostate tumors	HG-U133A, HG-U133Plus2.0, HG-U95B, HG-U95C	GSE3325, GSE6919 (HG-U95B, HG-U95C), GSE7930
**Metastasis group 28**	Primary melanoma VS. melanoma metastasis, poorly metastatic melanoma VS. highly metastatic melanoma	HG-U133A	GSE7929, GSE7956, GSE8401
**Metastasis group 29**	Normal melanocyte culture VS. culture of cutaneous metastasis of melanoma	HG-U133A, HG-U133B	GSE4840
**Metastasis group 30**	Primary melanoma VS. melanoma metastasis, poorly metastatic melanoma VS. highly metastatic melanoma, normal melanocyte culture VS. culture of cutaneous metastasis of melanoma	HG-U133A, HG-U133B	GSE4840, GSE7929, GSE7956, GSE8401
**Hypoxia group 1**	Hypoxia VS. normoxia	HG-U95Av2, HG-U133Plus2.0	GSE1056, GSE4086
