# Supplementary material for: Meta-analysis of archived DNA microarrays identifies genes regulated by hypoxia and involved in a metastatic phenotype in cancer cells
Source: BMC Cancer. 2010 Apr 30;10:176. doi: 10.1186/1471-2407-10-176 (PMC2880990; doi:10.1186/1471-2407-10-176)
Supplement: Additional file 8 — Meta-datasets. 14 meta-datasets were designed based on the experimental conditions. [file 1471-2407-10-176-S8.PDF]

	Experimental conditions	GeneChip models	Datasets
**Meta-dataset 1**	Primary tumor, normal tissue, poorly metastatic tissue VS. metastasis, highly metastatic tissue	HG-U133A	E-GEOD-1323, E-GEOD-2280, GSE2280, GSE2603, GSE4840 (HG-U133A), GSE7929, GSE7930, GSE7956, GSE8401
**Meta-dataset 2**	Primary tumor, poorly metastatic tissue VS. metastasis, highly metastatic tissue	HG-U133A	E-GEOD-1323, E-GEOD-2280, GSE2280, GSE2603, GSE7929, GSE7930, GSE7956, GSE8401
**Meta-dataset 3**	Primary tumor, normal tissue VS. metastasis	HG-U133A	E-GEOD-1323, E-GEOD-2280, GSE2280, GSE2603, GSE4840 (HG-U133A), GSE7929, GSE7956, GSE8401
**Meta-dataset 4**	Primary tumor VS. metastasis	HG-U133A	E-GEOD-1323, E-GEOD-2280, GSE2280, GSE2603, GSE4840 (HG-U133A), GSE7929, GSE7956, GSE8401
**Meta-dataset 5**	Primary tumor VS. metastasis	HG-U133A	E-GEOD-1323, E-GEOD-2280, GSE2280, GSE2603, GSE7929, GSE7956, GSE8401
**Meta-dataset 6**	Squamous cell carcinoma of the oral cavity VS. corresponding lymph node metastases	HG-U133A	E-GEOD-2280, GSE2280
**Meta-dataset 7**	Normal melanocyte culture, poorly metastatic melanoma, primary melanoma VS. culture of cutaneous metastasis of melanoma, highly metastatic melanoma, melanoma metastasis	HG-U133A	GSE4840 (HG-U133A), GSE7929, GSE7956, GSE8401
**Meta-dataset 8**	Poorly metastatic melanoma, primary melanoma VS. culture of cutaneous metastasis of melanoma, highly metastatic melanoma, melanoma metastasis	HG-U133A	GSE4840 (HG-U133A), GSE7929, GSE7956, GSE8401
**Meta-dataset 9**	Poorly metastatic melanoma, primary melanoma VS. highly metastatic melanoma, melanoma metastasis	HG-U133A	GSE7929, GSE7956, GSE8401
**Meta-dataset 10**	Primary tumor VS. metastasis	HG-U95Av2	E-MEXP-44 (HG-U95Av2), GSE6919 (HG-U95Av2)
**Meta-dataset 11**	Hypoxia VS. normoxia	HG-U95Av2	GSE1056
**Meta-dataset 12**	Primary tumor, normoxia VS. metastasis, hypoxia	HG-U133Plus2.0	GSE3325, GSE4086, GSE4843, GSE6369
**Meta-dataset 13**	Primary tumor VS. metastasis	HG-U133Plus2.0	GSE3325, GSE4843, GSE6369
**Meta-dataset 14**	Primary prostate cancer VS. metastases	HG-U133Plus2.0	GSE3325, GSE6369
