# Supplementary material for: Meta-analysis of archived DNA microarrays identifies genes regulated by hypoxia and involved in a metastatic phenotype in cancer cells
Source: BMC Cancer. 2010 Apr 30;10:176. doi: 10.1186/1471-2407-10-176 (PMC2880990; doi:10.1186/1471-2407-10-176)
Supplement: Additional file 9 — Table of references. This table reports the number of the references in the references section for all 183 genes of interest. These are the publications where those genes were shown to be involved in cancer (column 2), in metastasis (column 3) and/or in hypoxia (column 4). [file 1471-2407-10-176-S9.PDF]

Genes	Cancer	Metastasis	Hypoxia
ABAT	-	-	[1]
ADM	[2-5]	[6, 7]	[6]
ATF3	[8-10]	[11]	[12]
CDC6	[13]	[14]	-
CXCL1	[15, 16]	[17, 18]	-
HMGCS1	-	-	-
NR4A1	[19]	-	-
HOXA5	[20, 21]	-	-
ID2	[22-24]	[25]	[26]
ISL1	[27]	[27]	-
LMNB1	-	-	-
SERPINE1	[28-30]	[28-30]	[31]
TPSAB1	[32, 33]	-	[34]
ZFP36	[35, 36]	-	-
CCNE2	[37]	[38]	-
AKAP12	[39, 40]	[41]	[42]
STIP1	[43]	-	-
TIPARP	[44]	-	-
WSB1	[45]	[45]	-
TPSB2	[32, 33]	-	[34]
ASPM	[46]	[46]	-
RHOB	[47]	-	-
CALD1	[48]	-	-
CAPG	[49]	[50]	-
CCNB1	[51, 52]	[53]	-
CKS2	[54]	[54]	-
CLU	[55]	-	[56]
COL9A2	-	-	-
CTGF	[57]	-	[58]
DDB2	[59, 60]	-	-
DUSP1	[61]	[62]	-
DUSP8	-	-	-
DUT	-	-	-
ERBB3	[63]	[64]	-
GAPDH	-	-	-
HSPB1	[65, 66]	-	-
IDI1	[67, 68]	-	-
SP110	-	-	-
IGF2	[69]	-	[70]
ILF3	[71]	-	[71]
JUNB	[72]	[73]	-
LAMA3	[74]	[75]	-
LAMP1	-	-	-
TM4SF1	[76, 77]	[76, 77]	-
NFX1	-	-	-
PDK1	[78, 79]	-	[80]
PGK1	[81]	[82]	-
PHF2	-	-	-
MAPK1	-	-	-
MAP2K1	[83, 84]	-	[85]
RIT1	[86]	-	-
SCD	-	-	-
STAT1	[87]	-	-
TSPYL1	-	-	-
TUBB2A	-	-	-
VEGFA	[88]	-	[88]
PIR	[89]	[89]	-
BHLHB2	[90]	[90]	[91]
TP63	[92]	[93]	-
IER3	[94]	-	[95]
TUBB3	[96]	[97]	[98]
TUBB4	-	-	-
TUBB2C	-	-	-
DDX17	[99]	[100]	-
PSIP1	[101]	-	-
CARHSP1	-	-	-
UHRF1	[102]	-	-
ERO1L	-	-	[103-105]
SMOX	[106]	-	-
DDIT4	[107]	-	[107]
ZCCHC2	-	-	-

ZNF532	-	-	-
ASF1B	-	-	-
MLF1IP	[108, 109]	-	-
CYBRD1	[110, 111]	-	-
TUBB2B	-	-	-
ACTC1	-	-	-
ACTG2	-	-	-
ACYP2	-	-	-
ALOX15B	[112]	-	-
APP	-	-	-
BDH1	-	-	-
KLF9	[113]	-	-
C7	-	-	-
COL12A1	[114]	-	-
CTSB	[115]	-	-
DES	-	-	-
E2F6	[116]	-	-
PHC1	-	-	-
EIF2S3	-	-	-
FHL1	[117]	[117]	-
FLNC	-	-	-
FOS	[118]	-	-
GSTA4	-	-	-
GSTM1	[119, 120]	-	-
GSTM2	-	-	-
GSTM4	[121]	-	-
CFH	-	-	-
ID4	[122]	[122]	-
IDS	-	-	-
IGF1	[123, 124]	-	-
IGFBP1	-	-	-
ABLIM1	-	-	-
M6PR	[125]	-	-
MFAP4	-	-	-
MMP7	[126]	[126]	-
MNT	[127]	-	-
MYH11	[128, 129]	-	-
NFKBIA	[130, 131]	[130, 131]	-
CNTN3	-	-	-
PAX3	[132]	-	-
PRSS1	-	-	-
PRSS2	[133]	-	-
PRSS3	[134]	[134]	-
PRSS8	[135]	-	-
PTGDS	-	-	-
SPP1	[136]	[136]	-
TCEA3	-	-	-
PRDX2	[137]	[138]	-
TGM3	[139]	-	-
VSNL1	[140]	-	-
YWHAZ	[141]	-	-
SLC39A7	-	-	-
PDHX	-	-	-
SLC7A5	[142]	-	-
GPR109B	-	-	-
LITAF	-	-	-
GDF15	[143, 144]	[145]	-
HERPUD1	[146]	-	-
MYL9	-	-	-
STAG3	-	-	-
AKAP2	-	-	-
DDX42	-	-	-
PHLDA1	[147]	[147]	-
MTF2	-	-	-
TPX2	[148]	-	-
PRUNE2	-	-	-
MGRN1	-	-	-
TPSD1	-	-	-
MAFF	-	-	-
OSTF1	-	-	-
PRPF19	-	-	-
RNF141	-	-	-

IER3IP1	-	-	-
DTL	[149]	-	-
STAG3L1	-	-	-
PALMD	-	-	-
PRPF38B	-	-	-
NRIP3	-	-	-
PAK6	[150]	[150]	-
BHLHB3	[151]	-	[151]
PGBD5	-	-	-
FAM57A	[152]	-	-
RMI1	[153]	-	-
APOL6	[154]	-	-
KLHDC3	-	-	-
APCDD1	[155]	-	-
RBM24	-	-	-
GPR109A	-	-	-
STAG3L3	-	-	-
STAG3L2	-	-	-
PALM2-AKAP2	-	-	-
ARVCF	-	-	-
BCAT1	[156]	[157]	-
CFB	-	-	-
DHCR24	[158-160]	[158-160]	-
ETS2	[161, 162]	-	-
SFN	[163, 164]	-	-
HIST1H2AE	-	-	-
HSP90AA1	-	-	-
NDUFB6	-	-	-
PTHLH	[165]	[166]	-
RRM2	[167, 168]	-	-
TRAPPC2	-	-	-
SFRS6	-	-	-
TYMS	[169-171]	-	-
RGS20	[172]	[172]	-
SKAP2	[173]	-	-
SEDLP	-	-	-
ETHE1	-	-	-
LSM14A	-	-	-
ARID5B	-	-	-
LOC440258	-	-	-

1. Madl JE, Royer SM: **Glutamate dependence of GABA levels in neurons of hypoxic and hypoglycemic rat hippocampal slices.** *Neuroscience* 2000, **96:**657-664.
2. Nakamura M, Han B, Nunobiki O, Kakudo K: **Adrenomedullin: a tumor progression factor via angiogenic control.** *Curr Cancer Drug Targets* 2006, **6:**635-643.
3. Nikitenko LL, Fox SB, Kehoe S, Rees MC, Bicknell R: **Adrenomedullin and tumour angiogenesis.** *Br J Cancer* 2006, **94:**1-7.
4. Oehler MK, Fischer DC, Orlowska-Volk M, Herrle F, Kieback DG, Rees MC, Bicknell R: **Tissue and plasma expression of the angiogenic peptide adrenomedullin in breast cancer.** *Br J Cancer* 2003, **89:**1927-1933.
5. Zudaire E, Martinez A, Cuttitta F: **Adrenomedullin and cancer.** *Regul Pept* 2003, **112:**175-183.
6. Keleg S, Kayed H, Jiang X, Penzel R, Giese T, Buchler MW, Friess H, Kleeff J: **Adrenomedullin is induced by hypoxia and enhances pancreatic cancer cell invasion.** *Int J Cancer* 2007, **121:**21-32.
7. Ramachandran V, Arumugam T, Hwang RF, Greenson JK, Simeone DM, Logsdon CD: **Adrenomedullin is expressed in pancreatic cancer and stimulates cell proliferation and invasion in an autocrine manner via the adrenomedullin receptor, ADMR.** *Cancer Res* 2007, **67:**2666-2675.
8. Fan F, Jin S, Amundson SA, Tong T, Fan W, Zhao H, Zhu X, Mazzacurati L, Li X, Petrik KL, et al: **ATF3 induction following DNA damage is regulated by distinct**

signaling pathways and over-expression of ATF3 protein suppresses cells growth. *Oncogene* 2002, **21:**7488-7496.

9. Yan C, Wang H, Boyd DD: **ATF3 represses 72-kDa type IV collagenase (MMP-2) expression by antagonizing p53-dependent trans-activation of the collagenase promoter.** *J Biol Chem* 2002, **277:**10804-10812.
10. Yin X, Dewille JW, Hai T: **A potential dichotomous role of ATF3, an adaptive-response gene, in cancer development.** *Oncogene* 2008, **27:**2118-2127.
11. Ishiguro T, Nagawa H: **ATF3 gene regulates cell form and migration potential of HT29 colon cancer cells.** *Oncol Res* 2001, **12:**343-346.
12. Chen SC, Liu YC, Shyu KG, Wang DL: **Acute hypoxia to endothelial cells induces activating transcription factor 3 (ATF3) expression that is mediated via nitric oxide.** *Atherosclerosis* 2008, **201:**281-288.
13. Borlado LR, Mendez J: **CDC6: from DNA replication to cell cycle checkpoints and oncogenesis.** *Carcinogenesis* 2008, **29:**237-243.
14. Liontos M, Koutsami M, Sideridou M, Evangelou K, Kletsas D, Levy B, Kotsinas A, Nahum O, Zoumpourlis V, Kouloukoussa M, et al: **Deregulated overexpression of hCdt1 and hCdc6 promotes malignant behavior.** *Cancer Res* 2007, **67:**10899-10909.
15. Dhawan P, Richmond A: **Role of CXCL1 in tumorigenesis of melanoma.** *J Leukoc Biol* 2002, **72:**9-18.
16. Wang B, Hendricks DT, Wamunyokoli F, Parker MI: **A growth-related oncogene/CXC chemokine receptor 2 autocrine loop contributes to cellular proliferation in esophageal cancer.** *Cancer Res* 2006, **66:**3071-3077.
17. Li A, Varney ML, Singh RK: **Constitutive expression of growth regulated oncogene (gro) in human colon carcinoma cells with different metastatic potential and its role in regulating their metastatic phenotype.** *Clin Exp Metastasis* 2004, **21:**571-579.
18. Shintani S, Ishikawa T, Nonaka T, Li C, Nakashiro K, Wong DT, Hamakawa H: **Growth-regulated oncogene-1 expression is associated with angiogenesis and lymph node metastasis in human oral cancer.** *Oncology* 2004, **66:**316-322.
19. Mullican SE, Zhang S, Konopleva M, Ruvolo V, Andreeff M, Milbrandt J, Conneely OM: **Abrogation of nuclear receptors Nr4a3 and Nr4a1 leads to development of acute myeloid leukemia.** *Nat Med* 2007, **13:**730-735.
20. Raman V, Martensen SA, Reisman D, Evron E, Odenwald WF, Jaffee E, Marks J, Sukumar S: **Compromised HOXA5 function can limit p53 expression in human breast tumours.** *Nature* 2000, **405:**974-978.
21. Rhoads K, Arderiu G, Charboneau A, Hansen SL, Hoffman W, Boudreau N: **A role for Hox A5 in regulating angiogenesis and vascular patterning.** *Lymphat Res Biol* 2005, **3:**240-252.
22. Coppe JP, Itahana Y, Moore DH, Bennington JL, Desprez PY: **Id-1 and Id-2 proteins as molecular markers for human prostate cancer progression.** *Clin Cancer Res* 2004, **10:**2044-2051.
23. Gray MJ, Dallas NA, Van Buren G, Xia L, Yang AD, Somcio RJ, Gaur P, Mangala LS, Vivas-Mejia PE, Fan F, et al: **Therapeutic targeting of Id2 reduces growth of human colorectal carcinoma in the murine liver.** *Oncogene* 2008, **27:**7192-7200.
24. Itahana Y, Singh J, Sumida T, Coppe JP, Parrinello S, Bennington JL, Desprez PY: **Role of Id-2 in the maintenance of a differentiated and noninvasive phenotype in breast cancer cells.** *Cancer Res* 2003, **63:**7098-7105.
25. Tsunedomi R, Iizuka N, Tamesa T, Sakamoto K, Hamaguchi T, Somura H, Yamada M, Oka M: **Decreased ID2 promotes metastatic potentials of hepatocellular**

carcinoma by altering secretion of vascular endothelial growth factor. *Clin Cancer Res* 2008, **14:**1025-1031.

26. Lofstedt T, Jogi A, Sigvardsson M, Gradin K, Poellinger L, Pahlman S, Axelson H: **Induction of ID2 expression by hypoxia-inducible factor-1: a role in dedifferentiation of hypoxic neuroblastoma cells.** *J Biol Chem* 2004, **279:**39223-39231.
27. Schmitt AM, Riniker F, Anlauf M, Schmid S, Soltermann A, Moch H, Heitz PU, Kloppel G, Komminoth P, Perren A: **Islet 1 (Isl1) expression is a reliable marker for pancreatic endocrine tumors and their metastases.** *Am J Surg Pathol* 2008, **32:**420-425.
28. Binder BR, Mihaly J: **The plasminogen activator inhibitor "paradox" in cancer.** *Immunol Lett* 2008, **118:**116-124.
29. Dass K, Ahmad A, Azmi AS, Sarkar SH, Sarkar FH: **Evolving role of uPA/uPAR system in human cancers.** *Cancer Treat Rev* 2008, **34:**122-136.
30. Durand MK, Bodker JS, Christensen A, Dupont DM, Hansen M, Jensen JK, Kjelgaard S, Mathiasen L, Pedersen KE, Skeldal S, et al: **Plasminogen activator inhibitor-I and tumour growth, invasion, and metastasis.** *Thromb Haemost* 2004, **91:**438-449.
31. Dimova EY, Samoylenko A, Kietzmann T: **Oxidative stress and hypoxia: implications for plasminogen activator inhibitor-1 expression.** *Antioxid Redox Signal* 2004, **6:**777-791.
32. Nico B, Mangieri D, Crivellato E, Vacca A, Ribatti D: **Mast cells contribute to vasculogenic mimicry in multiple myeloma.** *Stem Cells Dev* 2008, **17:**19-22.
33. Ribatti D, Finato N, Crivellato E, Guidolin D, Longo V, Mangieri D, Nico B, Vacca A, Beltrami CA: **Angiogenesis and mast cells in human breast cancer sentinel lymph nodes with and without micrometastases.** *Histopathology* 2007, **51:**837-842.
34. Maxova H, Novotna J, Vajner L, Tomasova H, Vytasek R, Vizek M, Bacakova L, Valouskova V, Eliasova T, Herget J: **In vitro hypoxia increases production of matrix metalloproteinases and tryptase in isolated rat lung mast cells.** *Physiol Res* 2008, **57:**903-910.
35. Shimada H, Ichikawa H, Nakamura S, Katsu R, Iwasa M, Kitabayashi I, Ohki M: **Analysis of genes under the downstream control of the t(8;21) fusion protein AML1-MTG8: overexpression of the TIS11b (ERF-1, cMG1) gene induces myeloid cell proliferation in response to G-CSF.** *Blood* 2000, **96:**655-663.
36. Stoecklin G, Gross B, Ming XF, Moroni C: **A novel mechanism of tumor suppression by destabilizing AU-rich growth factor mRNA.** *Oncogene* 2003, **22:**3554-3561.
37. Butt AJ, Caldon CE, McNeil CM, Swarbrick A, Musgrove EA, Sutherland RL: **Cell cycle machinery: links with genesis and treatment of breast cancer.** *Adv Exp Med Biol* 2008, **630:**189-205.
38. Muller-Tidow C, Metzger R, Kugler K, Diederichs S, Idos G, Thomas M, Dockhorn-Dworniczak B, Schneider PM, Koeffler HP, Berdel WE, Serve H: **Cyclin E is the only cyclin-dependent kinase 2-associated cyclin that predicts metastasis and survival in early stage non-small cell lung cancer.** *Cancer Res* 2001, **61:**647-653.
39. Akakura S, Huang C, Nelson PJ, Foster B, Gelman IH: **Loss of the SSeCKS/Gravin/AKAP12 gene results in prostatic hyperplasia.** *Cancer Res* 2008, **68:**5096-5103.
40. Liu Y, Gao L, Gelman IH: **SSeCKS/Gravin/AKAP12 attenuates expression of proliferative and angiogenic genes during suppression of v-Src-induced oncogenesis.** *BMC Cancer* 2006, **6:**105.

41. Gelman IH, Gao L: **SSeCKS/Gravin/AKAP12 metastasis suppressor inhibits podosome formation via RhoA- and Cdc42-dependent pathways.** *Mol Cancer Res* 2006, **4:**151-158.
42. Choi YK, Kim JH, Kim WJ, Lee HY, Park JA, Lee SW, Yoon DK, Kim HH, Chung H, Yu YS, Kim KW: **AKAP12 regulates human blood-retinal barrier formation by downregulation of hypoxia-inducible factor-1alpha.** *J Neurosci* 2007, **27:**4472-4481.
43. Erlich RB, Kahn SA, Lima FR, Muras AG, Martins RA, Linden R, Chiarini LB, Martins VR, Moura Neto V: **STI1 promotes glioma proliferation through MAPK and PI3K pathways.** *Glia* 2007, **55:**1690-1698.
44. Katoh M, Katoh M: **Identification and characterization of human TIPARP gene within the CCNL amplicon at human chromosome 3q25.31.** *Int J Oncol* 2003, **23:**541-547.
45. Archange C, Nowak J, Garcia S, Moutardier V, Calvo EL, Dagorn JC, Iovanna JL: **The WSB1 gene is involved in pancreatic cancer progression.** *PLoS ONE* 2008, **3:**e2475.
46. Lin SY, Pan HW, Liu SH, Jeng YM, Hu FC, Peng SY, Lai PL, Hsu HC: **ASPM is a novel marker for vascular invasion, early recurrence, and poor prognosis of hepatocellular carcinoma.** *Clin Cancer Res* 2008, **14:**4814-4820.
47. Couderc B, Pradines A, Rafii A, Golzio M, Deviers A, Allal C, Berg D, Penary M, Teissie J, Favre G: **In vivo restoration of RhoB expression leads to ovarian tumor regression.** *Cancer Gene Ther* 2008, **15:**456-464.
48. Thorsen K, Sorensen KD, Brems-Eskildsen AS, Modin C, Gaustadnes M, Hein AM, Kruhoffer M, Laurberg S, Borre M, Wang K, et al: **Alternative splicing in colon, bladder, and prostate cancer identified by exon array analysis.** *Mol Cell Proteomics* 2008, **7:**1214-1224.
49. Partheen K, Levan K, Osterberg L, Claesson I, Fallenius G, Sundfeldt K, Horvath G: **Four potential biomarkers as prognostic factors in stage III serous ovarian adenocarcinomas.** *Int J Cancer* 2008, **123:**2130-2137.
50. Renz M, Betz B, Niederacher D, Bender HG, Langowski J: **Invasive breast cancer cells exhibit increased mobility of the actin-binding protein CapG.** *Int J Cancer* 2008, **122:**1476-1482.
51. Bjorck E, Ek S, Landgren O, Jerkeman M, Ehinger M, Bjorkholm M, Borrebaeck CA, Porwit-MacDonald A, Nordenskjold M: **High expression of cyclin B1 predicts a favorable outcome in patients with follicular lymphoma.** *Blood* 2005, **105:**2908-2915.
52. Wierinckx A, Auger C, Devauchelle P, Reynaud A, Chevallier P, Jan M, Perrin G, Fevre-Montange M, Rey C, Figarella-Branger D, et al: **A diagnostic marker set for invasion, proliferation, and aggressiveness of prolactin pituitary tumors.** *Endocr Relat Cancer* 2007, **14:**887-900.
53. de Haas T, Hasselt N, Troost D, Caron H, Popovic M, Zadravec-Zaletel L, Grajkowska W, Perek M, Osterheld MC, Ellison D, et al: **Molecular risk stratification of medulloblastoma patients based on immunohistochemical analysis of MYC, LDHB, and CCNB1 expression.** *Clin Cancer Res* 2008, **14:**4154-4160.
54. Kang MA, Kim JT, Kim JH, Kim SY, Kim YH, Yeom YI, Lee Y, Lee HG: **Upregulation of the cycline kinase subunit CKS2 increases cell proliferation rate in gastric cancer.** *J Cancer Res Clin Oncol* 2008.

55. Toffanin S, Daidone MG, Miodini P, De Cecco L, Gandellini P, Cappelletti V: **Clusterin: a potential target for improving response to antiestrogens.** *Int J Oncol* 2008, **33:**791-798.
56. Shin JK, Han KA, Kang MY, Kim YS, Park JK, Choi WJ, Lee SA, Lee JH, Choi WS, Paik WY: **Expression of clusterin in normal and preeclamptic placentas.** *J Obstet Gynaecol Res* 2008, **34:**473-479.
57. Bennewith KL, Huang X, Ham CM, Graves EE, Erler JT, Kambham N, Feazell J, Yang GP, Koong A, Giaccia AJ: **The role of tumor cell-derived connective tissue growth factor (CTGF/CCN2) in pancreatic tumor growth.** *Cancer Res* 2009, **69:**775-784.
58. Fuchshofer R, Yu AL, Teng HH, Strauss R, Kampik A, Welge-Lussen U: **Hypoxia/reoxygenation induces CTGF and PAI-1 in cultured human retinal pigment epithelium cells.** *Exp Eye Res* 2008.
59. Kattan Z, Marchal S, Brunner E, Ramacci C, Leroux A, Merlin JL, Domenjoud L, Dauca M, Becuwe P: **Damaged DNA binding protein 2 plays a role in breast cancer cell growth.** *PLoS ONE* 2008, **3:**e2002.
60. Xu QY, Gao Y, Liu Y, Yang WZ, Xu XY: **Identification of differential gene expression profiles of radioresistant lung cancer cell line established by fractionated ionizing radiation in vitro.** *Chin Med J (Engl)* 2008, **121:**1830-1837.
61. Wang Z, Zhou JY, Kanakapalli D, Buck S, Wu GS, Ravindranath Y: **High level of mitogen-activated protein kinase phosphatase-1 expression is associated with cisplatin resistance in osteosarcoma.** *Pediatr Blood Cancer* 2008, **51:**754-759.
62. Komiya Y, Kurabe N, Katagiri K, Ogawa M, Sugiyama A, Kawasaki Y, Tashiro F: **A novel binding factor of 14-3-3beta functions as a transcriptional repressor and promotes anchorage-independent growth, tumorigenicity, and metastasis.** *J Biol Chem* 2008, **283:**18753-18764.
63. Jackson-Fisher AJ, Bellinger G, Breindel JL, Tavassoli FA, Booth CJ, Duong JK, Stern DF: **ErbB3 is required for ductal morphogenesis in the mouse mammary gland.** *Breast Cancer Res* 2008, **10:**R96.
64. Lin SH, Cheng CJ, Lee YC, Ye X, Tsai WW, Kim J, Pasqualini R, Arap W, Navone NM, Tu SM, et al: **A 45-kDa ErbB3 secreted by prostate cancer cells promotes bone formation.** *Oncogene* 2008, **27:**5195-5203.
65. Kang SH, Kang KW, Kim KH, Kwon B, Kim SK, Lee HY, Kong SY, Lee ES, Jang SG, Yoo BC: **Upregulated HSP27 in human breast cancer cells reduces Herceptin susceptibility by increasing Her2 protein stability.** *BMC Cancer* 2008, **8:**286.
66. Oba M, Yano S, Shuto T, Suico MA, Eguma A, Kai H: **IFN-gamma down-regulates Hsp27 and enhances hyperthermia-induced tumor cell death in vitro and tumor suppression in vivo.** *Int J Oncol* 2008, **32:**1317-1324.
67. Bani MR, Nicoletti MI, Alkharouf NW, Ghilardi C, Petersen D, Erba E, Sausville EA, Liu ET, Giavazzi R: **Gene expression correlating with response to paclitaxel in ovarian carcinoma xenografts.** *Mol Cancer Ther* 2004, **3:**111-121.
68. Tabuchi Y, Ando H, Takasaki I, Feril LB, Jr., Zhao QL, Ogawa R, Kudo N, Tachibana K, Kondo T: **Identification of genes responsive to low intensity pulsed ultrasound in a human leukemia cell line Molt-4.** *Cancer Lett* 2007, **246:**149-156.
69. Suzuki H, Li Y, Dong X, Hassan MM, Abbruzzese JL, Li D: **Effect of insulin-like growth factor gene polymorphisms alone or in interaction with diabetes on the risk of pancreatic cancer.** *Cancer Epidemiol Biomarkers Prev* 2008, **17:**3467-3473.
70. Nakao K, Aoyama M, Fukuoka H, Fujita M, Miyazawa K, Asai K, Goto S: **IGF2 modulates the microenvironment for osteoclastogenesis.** *Biochem Biophys Res Commun* 2009, **378:**462-466.

71. Vumbaca F, Phoenix KN, Rodriguez-Pinto D, Han DK, Claffey KP: **Double-stranded RNA-binding protein regulates vascular endothelial growth factor mRNA stability, translation, and breast cancer angiogenesis.** *Mol Cell Biol* 2008, **28:**772-783.
72. Piechaczyk M, Farras R: **Regulation and function of JunB in cell proliferation.** *Biochem Soc Trans* 2008, **36:**864-867.
73. Konishi N, Shimada K, Nakamura M, Ishida E, Ota I, Tanaka N, Fujimoto K: **Function of JunB in transient amplifying cell senescence and progression of human prostate cancer.** *Clin Cancer Res* 2008, **14:**4408-4416.
74. Snijders AM, Schmidt BL, Fridlyand J, Dekker N, Pinkel D, Jordan RC, Albertson DG: **Rare amplicons implicate frequent deregulation of cell fate specification pathways in oral squamous cell carcinoma.** *Oncogene* 2005, **24:**4232-4242.
75. Hirai Y, Utsugi K, Takeshima N, Kawamata Y, Furuta R, Kitagawa T, Kawaguchi T, Hasumi K, Noda T: **Putative gene loci associated with carcinogenesis and metastasis of endocervical adenocarcinomas of uterus determined by conventional and array-based CGH.** *Am J Obstet Gynecol* 2004, **191:**1173-1182.
76. Kao YR, Shih JY, Wen WC, Ko YP, Chen BM, Chan YL, Chu YW, Yang PC, Wu CW, Roffler SR: **Tumor-associated antigen L6 and the invasion of human lung cancer cells.** *Clin Cancer Res* 2003, **9:**2807-2816.
77. Lekishvili T, Fromm E, Mujoomdar M, Berditchevski F: **The tumour-associated antigen L6 (L6-Ag) is recruited to the tetraspanin-enriched microdomains: implication for tumour cell motility.** *J Cell Sci* 2008, **121:**685-694.
78. Iorns E, Lord CJ, Ashworth A: **Parallel RNAi and compound screens identify the PDK1 pathway as a target for tamoxifen sensitization.** *Biochem J* 2009, **417:**361-370.
79. Peifer C, Alessi DR: **New anti-cancer role for PDK1 inhibitors: preventing resistance to tamoxifen.** *Biochem J* 2009, **417:**e5-7.
80. Vigano A, Ripamonti M, De Palma S, Capitanio D, Vasso M, Wait R, Lundby C, Cerretelli P, Gelfi C: **Proteins modulation in human skeletal muscle in the early phase of adaptation to hypobaric hypoxia.** *Proteomics* 2008, **8:**4668-4679.
81. Tang SW, Chang WH, Su YC, Chen YC, Lai YH, Wu PT, Hsu CI, Lin WC, Lai MK, Lin JY: **MYC pathway is activated in clear cell renal cell carcinoma and essential for proliferation of clear cell renal cell carcinoma cells.** *Cancer Lett* 2009, **273:**35-43.
82. Zieker D, Konigsrainer I, Traub F, Nieselt K, Knapp B, Schillinger C, Stirnkorb C, Fend F, Northoff H, Kupka S, et al: **PGK1 a potential marker for peritoneal dissemination in gastric cancer.** *Cell Physiol Biochem* 2008, **21:**429-436.
83. Marks JL, Gong Y, Chitale D, Golas B, McLellan MD, Kasai Y, Ding L, Mardis ER, Wilson RK, Solit D, et al: **Novel MEK1 mutation identified by mutational analysis of epidermal growth factor receptor signaling pathway genes in lung adenocarcinoma.** *Cancer Res* 2008, **68:**5524-5528.
84. Shama J, Garcia-Medina R, Pouyssegur J, Vial E: **Major contribution of MEK1 to the activation of ERK1/ERK2 and to the growth of LS174T colon carcinoma cells.** *Biochem Biophys Res Commun* 2008, **372:**845-849.
85. Bajpai AK, Blaskova E, Pakala SB, Zhao T, Glasgow WC, Penn JS, Johnson DA, Rao GN: **15(S)-HETE production in human retinal microvascular endothelial cells by hypoxia: Novel role for MEK1 in 15(S)-HETE induced angiogenesis.** *Invest Ophthalmol Vis Sci* 2007, **48:**4930-4938.

86. Hoshino M, Yoshimori T, Nakamura S: **Small GTPase proteins Rin and Rit Bind to PAR6 GTP-dependently and regulate cell transformation.** *J Biol Chem* 2005, **280:**22868-22874.
87. Klampfer L: **The role of signal transducers and activators of transcription in colon cancer.** *Front Biosci* 2008, **13:**2888-2899.
88. Goudar RK, Vlahovic G: **Hypoxia, angiogenesis, and lung cancer.** *Curr Oncol Rep* 2008, **10:**277-282.
89. Sun JY, Guo W, An J, Yuan Q, Zhao FK: **[Analysis of differentially expressed lung metastasis-associated proteins in adenoid cystic carcinoma cell lines].** *Zhonghua Kou Qiang Yi Xue Za Zhi* 2004, **39:**114-117.
90. Ehata S, Hanyu A, Hayashi M, Aburatani H, Kato Y, Fujime M, Saitoh M, Miyazawa K, Imamura T, Miyazono K: **Transforming growth factor-beta promotes survival of mammary carcinoma cells through induction of antiapoptotic transcription factor DEC1.** *Cancer Res* 2007, **67:**9694-9703.
91. Choi SM, Cho HJ, Cho H, Kim KH, Kim JB, Park H: **Stra13/DEC1 and DEC2 inhibit sterol regulatory element binding protein-1c in a hypoxia-inducible factor-dependent mechanism.** *Nucleic Acids Res* 2008, **36:**6372-6385.
92. Khokhar SK, Kommagani R, Kadakia MP: **Differential effects of p63 mutants on transactivation of p53 and/or p63 responsive genes.** *Cell Res* 2008, **18:**1061-1073.
93. Gu X, Coates PJ, Boldrup L, Nylander K: **p63 contributes to cell invasion and migration in squamous cell carcinoma of the head and neck.** *Cancer Lett* 2008, **263:**26-34.
94. Hu Y, Sun H, Drake J, Kittrell F, Abba MC, Deng L, Gaddis S, Sahin A, Baggerly K, Medina D, Aldaz CM: **From mice to humans: identification of commonly deregulated genes in mammary cancer via comparative SAGE studies.** *Cancer Res* 2004, **64:**7748-7755.
95. Tang Y, Pacary E, Freret T, Divoux D, Petit E, Schumann-Bard P, Bernaudin M: **Effect of hypoxic preconditioning on brain genomic response before and following ischemia in the adult mouse: identification of potential neuroprotective candidates for stroke.** *Neurobiol Dis* 2006, **21:**18-28.
96. Cicchillitti L, Penci R, Di Michele M, Filippetti F, Rotilio D, Donati MB, Scambia G, Ferlini C: **Proteomic characterization of cytoskeletal and mitochondrial class III beta-tubulin.** *Mol Cancer Ther* 2008, **7:**2070-2079.
97. Izutsu N, Maesawa C, Shibazaki M, Oikawa H, Shoji T, Sugiyama T, Masuda T: **Epigenetic modification is involved in aberrant expression of class III beta-tubulin, TUBB3, in ovarian cancer cells.** *Int J Oncol* 2008, **32:**1227-1235.
98. Raspaglio G, Filippetti F, Prislei S, Penci R, De Maria I, Cicchillitti L, Mozzetti S, Scambia G, Ferlini C: **Hypoxia induces class III beta-tubulin gene expression by HIF-1alpha binding to its 3' flanking region.** *Gene* 2008, **409:**100-108.
99. Somura H, Iizuka N, Tamesa T, Sakamoto K, Hamaguchi T, Tsunedomi R, Yamada-Okabe H, Sawamura M, Eramoto M, Miyamoto T, et al: **A three-gene predictor for early intrahepatic recurrence of hepatocellular carcinoma after curative hepatectomy.** *Oncol Rep* 2008, **19:**489-495.
100. Tan X, Zhai Y, Chang W, Hou J, He S, Lin L, Yu Y, Xu D, Xiao J, Ma L, et al: **Global analysis of metastasis-associated gene expression in primary cultures from clinical specimens of clear-cell renal-cell carcinoma.** *Int J Cancer* 2008, **123:**1080-1088.
101. Yokoyama A, Cleary ML: **Menin critically links MLL proteins with LEDGF on cancer-associated target genes.** *Cancer Cell* 2008, **14:**36-46.

102. Achour M, Jacq X, Ronde P, Alhosin M, Charlot C, Chataigneau T, Jeanblanc M, Macaluso M, Giordano A, Hughes AD, et al: **The interaction of the SRA domain of ICBP90 with a novel domain of DNMT1 is involved in the regulation of VEGF gene expression.** *Oncogene* 2008, **27:**2187-2197.
103. Baker KM, Chakravarthi S, Langton KP, Sheppard AM, Lu H, Bulleid NJ: **Low reduction potential of Ero1alpha regulatory disulphides ensures tight control of substrate oxidation.** *Embo J* 2008, **27:**2988-2997.
104. May D, Itin A, Gal O, Kalinski H, Feinstein E, Keshet E: **Ero1-L alpha plays a key role in a HIF-1-mediated pathway to improve disulfide bond formation and VEGF secretion under hypoxia: implication for cancer.** *Oncogene* 2005, **24:**1011-1020.
105. Yorita Y: **[Augmented expression of endoplasmic reticulum oxidoreductin 1-like (ERO1-L) under a hypoxic condition].** *Hokkaido Igaku Zasshi* 2005, **80:**87-93.
106. Hogarty MD, Norris MD, Davis K, Liu X, Evageliou NF, Hayes CS, Pawel B, Guo R, Zhao H, Sekyere E, et al: **ODC1 is a critical determinant of MYCN oncogenesis and a therapeutic target in neuroblastoma.** *Cancer Res* 2008, **68:**9735-9745.
107. Schneider A, Younis RH, Gutkind JS: **Hypoxia-induced energy stress inhibits the mTOR pathway by activating an AMPK/REDD1 signaling axis in head and neck squamous cell carcinoma.** *Neoplasia* 2008, **10:**1295-1302.
108. Hanissian SH, Teng B, Akbar U, Janjetovic Z, Zhou Q, Duntsch C, Robertson JH: **Regulation of myeloid leukemia factor-1 interacting protein (MLF1IP) expression in glioblastoma.** *Brain Res* 2005, **1047:**56-64.
109. Suzuki H, Arakawa Y, Ito M, Saito S, Takeda N, Yamada H, Horiguchi-Yamada J: **MLF1-interacting protein is mainly localized in nucleolus through N-terminal bipartite nuclear localization signal.** *Anticancer Res* 2007, **27:**1423-1430.
110. Boult J, Roberts K, Brookes MJ, Hughes S, Bury JP, Cross SS, Anderson GJ, Spychal R, Iqbal T, Tselepis C: **Overexpression of cellular iron import proteins is associated with malignant progression of esophageal adenocarcinoma.** *Clin Cancer Res* 2008, **14:**379-387.
111. Brookes MJ, Hughes S, Turner FE, Reynolds G, Sharma N, Ismail T, Berx G, McKie AT, Hotchin N, Anderson GJ, et al: **Modulation of iron transport proteins in human colorectal carcinogenesis.** *Gut* 2006, **55:**1449-1460.
112. Subbarayan V, Xu XC, Kim J, Yang P, Hoque A, Sabichi AL, Llansa N, Mendoza G, Logothetis CJ, Newman RA, et al: **Inverse relationship between 15-lipoxygenase-2 and PPAR-gamma gene expression in normal epithelia compared with tumor epithelia.** *Neoplasia* 2005, **7:**280-293.
113. Kang L, Lu B, Xu J, Hu H, Lai M: **Downregulation of Kruppel-like factor 9 in human colorectal cancer.** *Pathol Int* 2008, **58:**334-338.
114. Storlazzi CT, Wozniak A, Panagopoulos I, Sciot R, Mandahl N, Mertens F, Debiec-Rychter M: **Rearrangement of the COL12A1 and COL4A5 genes in subungual exostosis: molecular cytogenetic delineation of the tumor-specific translocation t(X;6)(q13-14;q22).** *Int J Cancer* 2006, **118:**1972-1976.
115. Rose PP, Bogyo M, Moses AV, Fruh K: **Insulin-like growth factor II receptor-mediated intracellular retention of cathepsin B is essential for transformation of endothelial cells by Kaposi's sarcoma-associated herpesvirus.** *J Virol* 2007, **81:**8050-8062.
116. Yang WW, Wang ZH, Zhu Y, Yang HT: **E2F6 negatively regulates ultraviolet-induced apoptosis via modulation of BRCA1.** *Cell Death Differ* 2007, **14:**807-817.

117. Sakashita K, Mimori K, Tanaka F, Kamohara Y, Inoue H, Sawada T, Hirakawa K, Mori M: **Clinical significance of loss of Fhl1 expression in human gastric cancer.** *Ann Surg Oncol* 2008, **15:**2293-2300.
118. Durchdewald M, Guinea-Viniegra J, Haag D, Riehl A, Lichter P, Hahn M, Wagner EF, Angel P, Hess J: **Podoplanin is a novel fos target gene in skin carcinogenesis.** *Cancer Res* 2008, **68:**6877-6883.
119. Tiwawech D, Chindavijak S, Karalak A, Ishida T: **Real-time PCR assay for rapid detection of GSTM1 polymorphism in nasopharyngeal carcinoma patients.** *Asian Pac J Cancer Prev* 2008, **9:**233-237.
120. Traka M, Gasper AV, Melchini A, Bacon JR, Needs PW, Frost V, Chantry A, Jones AM, Ortori CA, Barrett DA, et al: **Broccoli consumption interacts with GSTM1 to perturb oncogenic signalling pathways in the prostate.** *PLoS ONE* 2008, **3:**e2568.
121. Liloglou T, Walters M, Maloney P, Youngson J, Field JK: **A T2517C polymorphism in the GSTM4 gene is associated with risk of developing lung cancer.** *Lung Cancer* 2002, **37:**143-146.
122. Noetzel E, Veeck J, Niederacher D, Galm O, Horn F, Hartmann A, Knuchel R, Dahl E: **Promoter methylation-associated loss of ID4 expression is a marker of tumour recurrence in human breast cancer.** *BMC Cancer* 2008, **8:**154.
123. Cironi L, Riggi N, Provero P, Wolf N, Suva ML, Suva D, Kindler V, Stamenkovic I: **IGF1 is a common target gene of Ewing's sarcoma fusion proteins in mesenchymal progenitor cells.** *PLoS ONE* 2008, **3:**e2634.
124. Reeves SG, Rich D, Meldrum CJ, Colyvas K, Kurzawski G, Suchy J, Lubinski J, Scott RJ: **IGF1 is a modifier of disease risk in hereditary non-polyposis colorectal cancer.** *Int J Cancer* 2008, **123:**1339-1343.
125. O'Gorman DB, Weiss J, Hettiaratchi A, Firth SM, Scott CD: **Insulin-like growth factor-II/mannose 6-phosphate receptor overexpression reduces growth of choriocarcinoma cells in vitro and in vivo.** *Endocrinology* 2002, **143:**4287-4294.
126. Zhang JQ, Wan YL, Liu YC, Wang X, Tang JQ, Wu T, Zhu J, Pan YS: **The FVIIa-tissue factor complex induces the expression of MMP7 in LOVO cells in vitro.** *Int J Colorectal Dis* 2008, **23:**971-978.
127. Hooker CW, Hurlin PJ: **Of Myc and Mnt.** *J Cell Sci* 2006, **119:**208-216.
128. Alhopuro P, Karhu A, Winqvist R, Waltering K, Visakorpi T, Aaltonen LA: **Somatic mutation analysis of MYH11 in breast and prostate cancer.** *BMC Cancer* 2008, **8:**263.
129. Sammalkorpi H, Alhopuro P, Niittymaki I, Orntoft TF, Hokland P, Karhu A, Aaltonen LA: **Mutation analysis of MYH11 in acute myeloid leukemia.** *Leuk Lymphoma* 2008, **49:**1829-1831.
130. Bu H, Rosdahl I, Sun XF, Zhang H: **Importance of polymorphisms in NF-kappaB1 and NF-kappaBIalpha genes for melanoma risk, clinicopathological features and tumor progression in Swedish melanoma patients.** *J Cancer Res Clin Oncol* 2007, **133:**859-866.
131. Gao J, Pfeifer D, He LJ, Qiao F, Zhang Z, Arbman G, Wang ZL, Jia CR, Carstensen J, Sun XF: **Association of NFKBIA polymorphism with colorectal cancer risk and prognosis in Swedish and Chinese populations.** *Scand J Gastroenterol* 2007, **42:**345-350.
132. Lesiak K, Sztiller-Sikorska M, Czyz M: **[Transcription factors in the development and progression of melanoma].** *Postepy Hig Med Dosw (Online)* 2007, **61:**576-595.
133. Vilen ST, Nyberg P, Hukkanen M, Sutinen M, Ylipalosaari M, Bjartell A, Paju A, Haaparanta V, Stenman UH, Sorsa T, Salo T: **Intracellular co-localization of**

trypsin-2 and matrix metalloprotease-9: possible proteolytic cascade of trypsin-2, MMP-9 and enterokinase in carcinoma. *Exp Cell Res* 2008, **314:**914-926.

134. Ghilardi C, Chiorino G, Dossi R, Nagy Z, Giavazzi R, Bani M: **Identification of novel vascular markers through gene expression profiling of tumor-derived endothelium.** *BMC Genomics* 2008, **9:**201.

135. Verghese GM, Gutknecht MF, Caughey GH: **Prostasin regulates epithelial monolayer function: cell-specific Gpld1-mediated secretion and functional role for GPI anchor.** *Am J Physiol Cell Physiol* 2006, **291:**C1258-1270.

136. Oler G, Camacho CP, Hojaij FC, Michaluart P, Jr., Riggins GJ, Cerutti JM: **Gene expression profiling of papillary thyroid carcinoma identifies transcripts correlated with BRAF mutational status and lymph node metastasis.** *Clin Cancer Res* 2008, **14:**4735-4742.

137. Furuta J, Nobeyama Y, Umebayashi Y, Otsuka F, Kikuchi K, Ushijima T: **Silencing of Peroxiredoxin 2 and aberrant methylation of 33 CpG islands in putative promoter regions in human malignant melanomas.** *Cancer Res* 2006, **66:**6080-6086.

138. Carta F, Demuro PP, Zanini C, Santona A, Castiglia D, D'Atri S, Ascierto PA, Napolitano M, Cossu A, Tadolini B, et al: **Analysis of candidate genes through a proteomics-based approach in primary cell lines from malignant melanomas and their metastases.** *Melanoma Res* 2005, **15:**235-244.

139. Liu W, Yu ZC, Cao WF, Ding F, Liu ZH: **Functional studies of a novel oncogene TGM3 in human esophageal squamous cell carcinoma.** *World J Gastroenterol* 2006, **12:**3929-3932.

140. Fu J, Fong K, Bellacosa A, Ross E, Apostolou S, Bassi DE, Jin F, Zhang J, Cairns P, Ibanez de Caceres I, et al: **VILIP-1 downregulation in non-small cell lung carcinomas: mechanisms and prediction of survival.** *PLoS ONE* 2008, **3:**e1698.

141. Ralhan R, Desouza LV, Matta A, Chandra Tripathi S, Ghanny S, Datta Gupta S, Bahadur S, Siu KW: **Discovery and verification of head-and-neck cancer biomarkers by differential protein expression analysis using iTRAQ labeling, multidimensional liquid chromatography, and tandem mass spectrometry.** *Mol Cell Proteomics* 2008, **7:**1162-1173.

142. Kim CS, Cho SH, Chun HS, Lee SY, Endou H, Kanai Y, Kim do K: **BCH, an inhibitor of system L amino acid transporters, induces apoptosis in cancer cells.** *Biol Pharm Bull* 2008, **31:**1096-1100.

143. Scrideli CA, Carlotti CG, Jr., Okamoto OK, Andrade VS, Cortez MA, Motta FJ, Lucio-Eterovic AK, Neder L, Rosemberg S, Oba-Shinjo SM, et al: **Gene expression profile analysis of primary glioblastomas and non-neoplastic brain tissue: identification of potential target genes by oligonucleotide microarray and real-time quantitative PCR.** *J Neurooncol* 2008, **88:**281-291.

144. Wan F, Miao X, Quraishi I, Kennedy V, Creek KE, Pirisi L: **Gene expression changes during HPV-mediated carcinogenesis: a comparison between an in vitro cell model and cervical cancer.** *Int J Cancer* 2008, **123:**32-40.

145. Zimmers TA, Jin X, Gutierrez JC, Acosta C, McKillop IH, Pierce RH, Koniaris LG: **Effect of in vivo loss of GDF-15 on hepatocellular carcinogenesis.** *J Cancer Res Clin Oncol* 2008, **134:**753-759.

146. Joo JH, Liao G, Collins JB, Grissom SF, Jetten AM: **Farnesol-induced apoptosis in human lung carcinoma cells is coupled to the endoplasmic reticulum stress response.** *Cancer Res* 2007, **67:**7929-7936.

147. Neef R, Kuske MA, Prols E, Johnson JP: **Identification of the human PHLDA1/TDAG51 gene: down-regulation in metastatic melanoma contributes to apoptosis resistance and growth deregulation.** *Cancer Res* 2002, **62:**5920-5929.
148. Xia F, Canovas PM, Guadagno TM, Altieri DC: **A survivin-ran complex regulates spindle formation in tumor cells.** *Mol Cell Biol* 2008, **28:**5299-5311.
149. Ueki T, Nishidate T, Park JH, Lin ML, Shimo A, Hirata K, Nakamura Y, Katagiri T: **Involvement of elevated expression of multiple cell-cycle regulator, DTL/RAMP (denticleless/RA-regulated nuclear matrix associated protein), in the growth of breast cancer cells.** *Oncogene* 2008, **27:**5672-5683.
150. Kaur R, Yuan X, Lu ML, Balk SP: **Increased PAK6 expression in prostate cancer and identification of PAK6 associated proteins.** *Prostate* 2008, **68:**1510-1516.
151. Nakamura H, Tanimoto K, Hiyama K, Yunokawa M, Kawamoto T, Kato Y, Yoshiga K, Poellinger L, Hiyama E, Nishiyama M: **Human mismatch repair gene, MLH1, is transcriptionally repressed by the hypoxia-inducible transcription factors, DEC1 and DEC2.** *Oncogene* 2008, **27:**4200-4209.
152. Pan DN, Li JJ, Wei L, Yao M, Wan DF, Gu JR: **Inhibitory effect of CT120B, an alternative splice variant of CT120A, on lung cancer cell growth.** *Acta Biochim Biophys Sin (Shanghai)* 2005, **37:**588-592.
153. Broberg K, Hoglund M, Gustafsson C, Bjork J, Ingvar C, Albin M, Olsson H: **Genetic variant of the human homologous recombination-associated gene RMI1 (S455N) impacts the risk of AML/MDS and malignant melanoma.** *Cancer Lett* 2007, **258:**38-44.
154. Liu Z, Lu H, Jiang Z, Pastuszyn A, Hu CA: **Apolipoprotein l6, a novel proapoptotic Bcl-2 homology 3-only protein, induces mitochondria-mediated apoptosis in cancer cells.** *Mol Cancer Res* 2005, **3:**21-31.
155. Takahashi M, Fujita M, Furukawa Y, Hamamoto R, Shimokawa T, Miwa N, Ogawa M, Nakamura Y: **Isolation of a novel human gene, APCDD1, as a direct target of the beta-Catenin/T-cell factor 4 complex with probable involvement in colorectal carcinogenesis.** *Cancer Res* 2002, **62:**5651-5656.
156. Zhou W, Feng X, Li H, Wang L, Li H, Zhu B, Zhang H, Yao K, Ren C: **Functional evidence for a nasopharyngeal carcinoma-related gene BCAT1 located at 12p12.** *Oncol Res* 2007, **16:**405-413.
157. Yoshikawa R, Yanagi H, Shen CS, Fujiwara Y, Noda M, Yagyu T, Gega M, Oshima T, Yamamura T, Okamura H, et al: **ECA39 is a novel distant metastasis-related biomarker in colorectal cancer.** *World J Gastroenterol* 2006, **12:**5884-5889.
158. Bonaccorsi L, Luciani P, Nesi G, Mannucci E, Deledda C, Dichiara F, Paglierani M, Rosati F, Masieri L, Serni S, et al: **Androgen receptor regulation of the seladin-1/DHCR24 gene: altered expression in prostate cancer.** *Lab Invest* 2008, **88:**1049-1056.
159. Di Stasi D, Vallacchi V, Campi V, Ranzani T, Daniotti M, Chiodini E, Fiorentini S, Greeve I, Prinetti A, Rivoltini L, et al: **DHCR24 gene expression is upregulated in melanoma metastases and associated to resistance to oxidative stress-induced apoptosis.** *Int J Cancer* 2005, **115:**224-230.
160. Hendriksen PJ, Dits NF, Kokame K, Veldhoven A, van Weerden WM, Bangma CH, Trapman J, Jenster G: **Evolution of the androgen receptor pathway during progression of prostate cancer.** *Cancer Res* 2006, **66:**5012-5020.
161. De la Houssaye G, Vieira V, Masson C, Beermann F, Dufier JL, Menasche M, Abitbol M: **ETS-1 and ETS-2 are upregulated in a transgenic mouse model of pigmented ocular neoplasm.** *Mol Vis* 2008, **14:**1912-1928.

162. Xu D, Dwyer J, Li H, Duan W, Liu JP: **Ets2 maintains hTERT gene expression and breast cancer cell proliferation by interacting with c-Myc.** *J Biol Chem* 2008, **283:**23567-23580.
163. Kovacheva VP, Davison JM, Mellott TJ, Rogers AE, Yang S, O'Brien MJ, Blusztajn JK: **Raising gestational choline intake alters gene expression in DMBA-evoked mammary tumors and prolongs survival.** *Faseb J* 2009, **23:**1054-1063.
164. Wang Z, Trope CG, Suo Z, Troen G, Yang G, Nesland JM, Holm R: **The clinicopathological and prognostic impact of 14-3-3 sigma expression on vulvar squamous cell carcinomas.** *BMC Cancer* 2008, **8:**308.
165. Ye H, Yu T, Temam S, Ziober BL, Wang J, Schwartz JL, Mao L, Wong DT, Zhou X: **Transcriptomic dissection of tongue squamous cell carcinoma.** *BMC Genomics* 2008, **9:**69.
166. Nguyen ST, Hasegawa S, Tsuda H, Tomioka H, Ushijima M, Noda M, Omura K, Miki Y: **Identification of a predictive gene expression signature of cervical lymph node metastasis in oral squamous cell carcinoma.** *Cancer Sci* 2007, **98:**740-746.
167. Boukovinas I, Papadaki C, Mendez P, Taron M, Mavroudis D, Koutsopoulos A, Sanchez-Ronco M, Sanchez JJ, Trypaki M, Staphopoulos E, et al: **Tumor BRCA1, RRM1 and RRM2 mRNA expression levels and clinical response to first-line gemcitabine plus docetaxel in non-small-cell lung cancer patients.** *PLoS ONE* 2008, **3:**e3695.
168. Duxbury MS, Whang EE: **RRM2 induces NF-kappaB-dependent MMP-9 activation and enhances cellular invasiveness.** *Biochem Biophys Res Commun* 2007, **354:**190-196.
169. Aarhus M, Bruland O, Bredholt G, Lybaek H, Husebye ES, Krossnes BK, Vedeler C, Wester K, Lund-Johansen M, Knappskog PM: **Microarray analysis reveals down-regulation of the tumour suppressor gene WWOX and up-regulation of the oncogene TYMS in intracranial sporadic meningiomas.** *J Neurooncol* 2008, **88:**251-259.
170. Kim HN, Lee IK, Kim YK, Tran HT, Yang DH, Lee JJ, Shin MH, Park KS, Shin MG, Choi JS, Kim HJ: **Association between folate-metabolizing pathway polymorphism and non-Hodgkin lymphoma.** *Br J Haematol* 2008, **140:**287-294.
171. Zhai X, Gao J, Hu Z, Tang J, Qin J, Wang S, Wang X, Jin G, Liu J, Chen W, et al: **Polymorphisms in thymidylate synthase gene and susceptibility to breast cancer in a Chinese population: a case-control analysis.** *BMC Cancer* 2006, **6:**138.
172. Riker AI, Enkemann SA, Fodstad O, Liu S, Ren S, Morris C, Xi Y, Howell P, Metge B, Samant RS, et al: **The gene expression profiles of primary and metastatic melanoma yields a transition point of tumor progression and metastasis.** *BMC Med Genomics* 2008, **1:**13.
173. Harada T, Chelala C, Bhakta V, Chaplin T, Caulee K, Baril P, Young BD, Lemoine NR: **Genome-wide DNA copy number analysis in pancreatic cancer using high-density single nucleotide polymorphism arrays.** *Oncogene* 2008, **27:**1951-1960.
