# Supplementary figures and images for: Meta-analysis of archived DNA microarrays identifies genes regulated by hypoxia and involved in a metastatic phenotype in cancer cells
Source: BMC Cancer. 2010 Apr 30;10:176. doi: 10.1186/1471-2407-10-176 (PMC2880990; doi:10.1186/1471-2407-10-176)

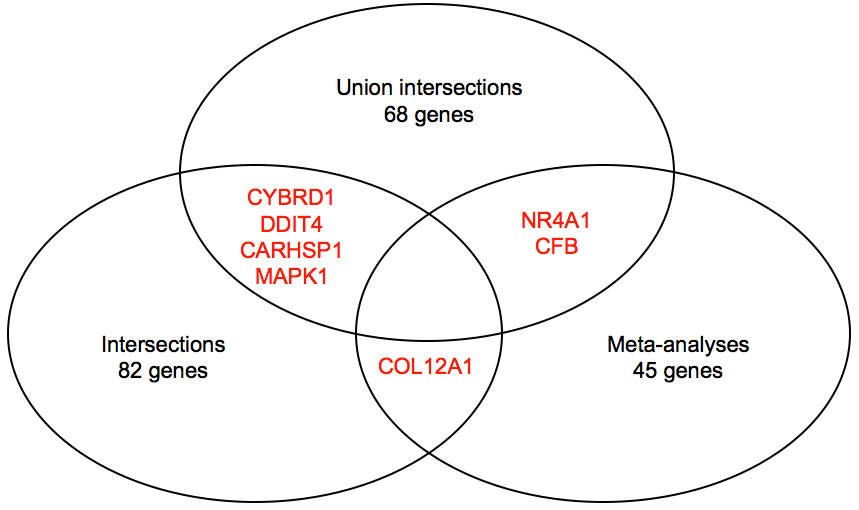

Supplement: Additional file 10 — Venn's diagram for the prostate datasets. The 6 prostate specific datasets (or sub-datasets) were used to run two intersections, two union intersections and one meta-analysis. These three approaches provided 87, 74 and 48 genes respectively. A Venn's diagram was then generated using these data. [file 1471-2407-10-176-S10.PNG]

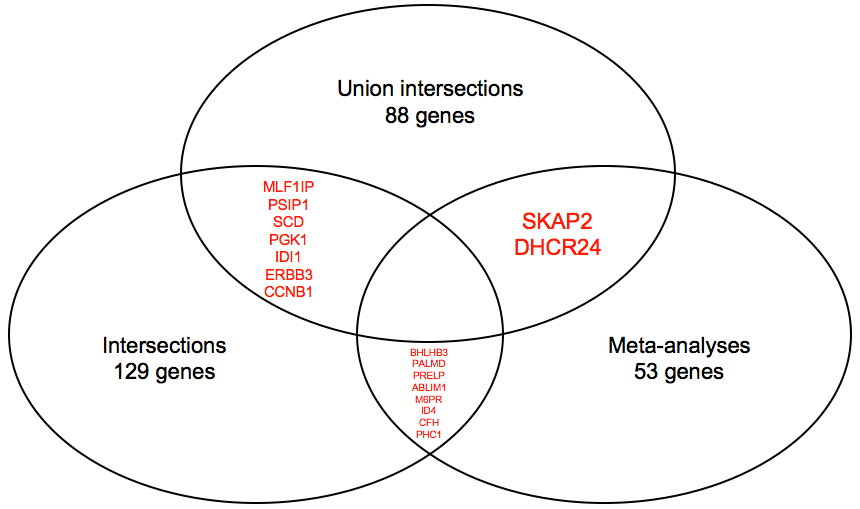

Supplement: Additional file 11 — Venn's diagram for the melanoma datasets. The 6 melanoma specific datasets (or sub-datasets) were used to run three intersections, three union intersections and three meta-analyses. These three approaches provided 144, 97 and 63 genes respectively. A Venn's diagram was then generated using these data. [file 1471-2407-10-176-S11.PNG]
